# Multiplex interrogation of the NK cell signalome reveals global downregulation of CD16 signaling during lentivirus infection through an IL-18/ADAM17-dependent mechanism

**DOI:** 10.1371/journal.ppat.1011629

**Published:** 2023-09-05

**Authors:** Sho Sugawara, Brady Hueber, Griffin Woolley, Karen Terry, Kyle Kroll, Cordelia Manickam, Daniel R. Ram, Lishomwa C. Ndhlovu, Paul Goepfert, Stephanie Jost, R. Keith Reeves

**Affiliations:** 1 Division of Innate and Comparative Immunology, Center for Human Systems Immunology, Duke University School of Medicine, Durham, North Carolina, United States of America; 2 Department of Surgery, Duke University, Durham, North Carolina, United States of America; 3 Center for Virology and Vaccine Research, Beth Israel Deaconess Medical Center, Harvard Medical School, Boston, Massachusetts, United States of America; 4 Department of Medicine, Division of Infectious Diseases, Weill Cornell Medicine, New York City, New York, United States of America; 5 University of Alabama at Birmingham, Birmingham, Alabama, United States of America; University of Wisconsin, UNITED STATES

## Abstract

Despite their importance, natural killer (NK) cell responses are frequently dysfunctional during human immunodeficiency virus-1 (HIV-1) and simian immunodeficiency virus (SIV) infections, even irrespective of antiretroviral therapies, with poorly understood underlying mechanisms. NK cell surface receptor modulation in lentivirus infection has been extensively studied, but a deeper interrogation of complex cell signaling is mostly absent, largely due to the absence of any comprehensive NK cell signaling assay. To fill this knowledge gap, we developed a novel multiplex signaling analysis to broadly assess NK cell signaling. Using this assay, we elucidated that NK cells exhibit global signaling reduction from CD16 both in people living with HIV-1 (PLWH) and SIV-infected rhesus macaques. Intriguingly, antiretroviral treatment did not fully restore diminished CD16 signaling in NK cells from PLWH. As a putative mechanism, we demonstrated that NK cells increased surface ADAM17 expression via elevated plasma IL-18 levels during HIV-1 infection, which in turn reduced surface CD16 downregulation. We also illustrated that CD16 expression and signaling can be restored by ADAM17 perturbation. In summary, our multiplex NK cell signaling analysis delineated unique NK cell signaling perturbations specific to lentiviral infections, resulting in their dysfunction. Our analysis also provides mechanisms that will inform the restoration of dysregulated NK cell functions, offering potential insights for the development of new NK cell-based immunotherapeutics for HIV-1 disease.

## Author summary

Natural killer (NK) cells exert critical innate effector responses against human immunodeficiency virus-1 (HIV-1) and simian immunodeficiency virus (SIV) infections, but their functions are often dysregulated during chronic lentiviral infection with understudied mechanisms. Specifically, the effect on NK cell signaling linked to this immune dysfunction has not been comprehensively elucidated. To fill the gap in knowledge, we developed a novel multiplex signaling assay for NK cells and applied it to NK cells from people living with HIV-1 (PLWH) or SIV-infected rhesus macaques (RM). We illustrated the signaling activation downstream of CD16, a critical receptor for NK cell function, is systemically downregulated in NK cells in chronic lentiviral infection regardless of antiretroviral treatment, which is mediated by inflammatory responses triggered by HIV-1/SIV infection. Taken together, we demonstrated global CD16 signaling downregulation in NK cells in HIV-1 and SIV infection using our novel multiplex signaling assay and elucidated the putative mechanism of impaired NK cell activities. These data are beneficial to further advance NK cell-based immunotherapeutics for an HIV-1 cure.

## Introduction

Natural killer (NK) cells are important innate effector cells for limiting human immunodeficiency virus-1 (HIV-1) and simian immunodeficiency virus (SIV) transmission and pathogenesis [1–6]. Lack of HIV-1 disease progression has been associated with specific killer immunoglobulin-like receptor (KIR) haplotypes [7–10], and robust NK cell antibody-dependent cellular cytotoxicity (ADCC) responses are associated with more successful protection following HIV-1 vaccination [1,11,12]. Similarly, in SIV infections, multifaceted NK cell activities are key contributors for SIV control [13–15]. Robust NK cell activities were frequently observed in animals with low SIV viral load (VL) and specific KIR genotypes are associated with SIV control similar to people living with HIV-1 (PLWH) [13]. Our recent NK cell depletion study in rhesus macaques (RM) illustrated that NK cells are crucial for mitigating SIV transmission and dissemination during the acute phase of infection [5].

Despite their significance, NK cell responses are frequently dysregulated in HIV-1/SIV infections. Regardless of antiretrovirals (ART), PLWH accumulate CD56^-^CD16^+^ NK cell subsets that elicit weaker cytokine production and cytotoxicity [16–20]. Presumably due to the increase in this subset, PLWH NK cells exhibit diminished cytolytic responses and ADCC [21,22]. NK cells from PLWH also induce less maturation of autologous dendritic cells because of their weaker interaction [23]. In agreement with what has been reported in HIV-1 infection, NK cell activities are also impaired in SIV-infected RM. LaBonte et al. described that NK cells gradually diminish cytokine production following SIV infection with reduced NKG2 mRNA expression [24]. Besides becoming less functional, NK cells exhibit altered trafficking capacity during lentiviral infections. Increased NK cell homing to the gut was characterized in SIV infection, and gut NK cells demonstrated diminished regulatory activity and enhanced cytotoxicity [25,26]. Consequently, these cytolytic NK cells perturb other gut immune cells including depletion of innate lymphoid cells [26]. Conversely, in pathogenic SIV infection, NK cells do not migrate to the lymph nodes due to the loss of the homing receptor expression [27]. Our recent studies in PLWH cohorts further elucidated the inverse correlation between HIV-1 VL and gut homing receptor α4β7-expressing NK cells, whereas the lymph node trafficking marker CCR7 expression positively associated with HIV-1 VL [6], implying a change in NK cell homing also in HIV-1 infection. In addition to homing receptors, the dysregulation of both activating and inhibitory receptors has been shown in PLWH and SIV-infected RM by multiple groups [14,16–20,24,28]. Whereas the alteration of surface receptor expression profiles has been extensively investigated in HIV-1/SIV infection, the impact on downstream intracellular signaling remains poorly studied [29].

NK cells integrate signals from activating and inhibitory receptors to orchestrate a variety of functions [29]. Engagement of activating receptors CD16 (Fcγ receptor IIIa), NKp46, and NKp30 induces phosphorylation of immunotyrosine activation motif (ITAM)-bearing adaptor molecules Fc receptor γ chain (FcRγ) and CD3 ζ chain (CD3ζ) [30–36]. Other activating receptors such as NKG2C, NKp44, and the majority of activating KIRs are associated with another ITAM-harboring molecule DAP12 to relay activation signals [37–39]. The signals from these receptors then converge into ITAM-based signaling pathways including Syk, ZAP70, and LAT phosphorylation, followed by activation of MAP kinase (JNK), PLCγ1, Vav-2, and Vav-3 proteins [31–36,40–44]. Conversely, NKG2D induces its unique signaling using the non-ITAM-bearing adaptor molecule DAP10 and activation of distinct kinases and signaling molecules including PI3K, PLCγ2, Vav-1, and NF-κB, is independent from ITAM-based pathways [45,46]. These activating signals are further enhanced by engagement of co-receptors including CD2, 2B4, and DNAM-1 [45,47–49]. To inhibit NK cell functions, inhibitory receptors such as NKG2A and inhibitory KIRs dephosphorylate signaling molecules by recruiting phosphatases SHP-1 and SHIP-1 upon ligand engagement [50,51].

NK cell signaling activation has been traditionally monitored by classical techniques including western blotting and phosphoflow [29]. While these assays are highly informative, they can quantify only a few phospho (p)-proteins at a time, which results in incomplete understanding of multidimensional signaling pathways [29,42,52,53]. To address this problem, multiplex signaling analyses have recently been developed for other cell types, including T cells [29,54] and leukemic cells [55–57]. Findings from these types of assays are beneficial for screening novel targets for immunotherapeutics and drug development [58,59]. Therefore, to fill the gap in knowledge between the modulation of NK cell surface receptor expression and subsequent intracellular signaling, we established a Luminex-based multiplex signaling assay to comprehensively analyze signaling activation in human and RM NK cells. We further applied this multiplex signaling assay to NK cells from PLWH and chronically SIV-infected RM to better understand the impact of altered signaling on function during chronic lentivirus infections.

## Results

### Novel multiplex platform captures multiple phosphorylation signaling events simultaneously in human and RM NK cells

**Fig 1:**
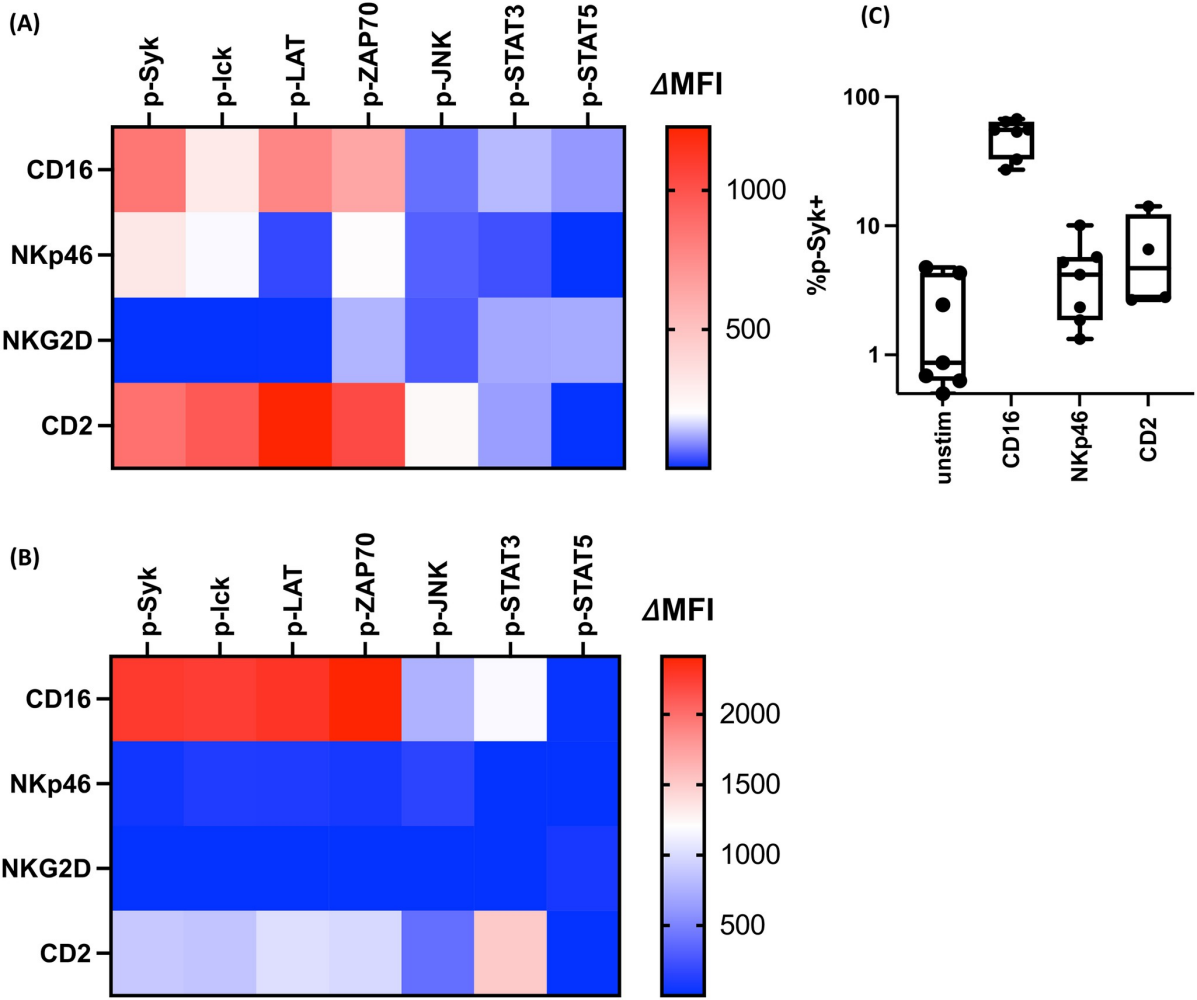
Human and RM NK cell multiplex signaling analysis is consistent with conventional assays. NK cells from healthy human donors **(A)** and experimentally naïve RM **(B)** were enriched from PBMCs and stimulated with anti-CD16 (A; n = 15, B; n = 6), anti-NKp46 (A; n = 13, B; n = 6), anti-NKG2D (A; n = 15, B; n = 6), or anti-CD2 (A; n = 13, B; n = 6) antibodies. Cells were then lysed, and total protein was collected. The levels of phosphorylated proteins were quantified by Luminex assay. Shown are the summary heatmaps where the MFI of phosphorylated proteins were normalized by the amount of GAPDH protein in each sample. ΔMFI was calculated by background subtraction using samples crosslinked with anti-β2M antibodies. Signals lower than β2M-stimulated samples are depicted as ΔMFI of 1. **(C)** Human PBMC were stimulated with anti-CD16 (n = 7), anti-NKp46 (n = 7), and anti-CD2 antibodies (n = 4), or left unstimulated (n = 7). Following stimulation, cells were stained and analyzed by flow cytometry. The summary dot plots for % p-Syk^+^ cells were depicted. Each dot represents a data point from a different individual.

We first stimulated human NK cells from peripheral blood mononuclear cells (PBMC) derived from individuals without HIV-1 (HIV^-^) infection through multiple activating receptors including CD16 (Fc receptor), NKp46 (natural cytotoxicity receptor family), NKG2D (NKG2 family of C-type lectin-like receptors), and CD2 (co-receptor), and tested our composite Luminex signaling assay to assess the activation of several canonical signaling cascades, including ITAM-based signaling (p-Syk, p-lck, p-LAT, and p-ZAP70), STAT signaling (p-STAT3 and p-STAT5), MAP kinase (MAPK) signaling (p-JNK, p-p38, p-ERK, and p-CREB), NF-κB signaling (p-NF-κB), and mTOR signaling (p-Akt and p-p70S6K). CD16 and CD2 crosslinking triggered the robust activation of ITAM-based signaling, MAPK, and STAT signaling (Fig 1A). Although signals were more modest, NKp46 stimulation also resulted in low-level activation as expected. Consistent with previous reports from other groups [46,60], NKG2D crosslinking did not induce ITAM-based signaling, but STAT3 pathways were somewhat upregulated.

Next, we tested naïve blood NK cells derived from uninfected RM to confirm the compatibility of our Luminex assay with RM. As anticipated, the Luminex signaling platform was able to detect the phosphorylation of the same signaling molecules from RM NK cells (Fig 1B) in a comparable fashion. We also observed analogous signaling activation profiles between human and RM NK cells, whereby CD16 crosslinking triggered the strongest ITAM-based, MAPK, and STAT signaling, while NKp46 and CD2 stimulation induced more moderate responses (Fig 1A and 1B).

To confirm that the multiplex signaling assay produced comparable results compared to more traditional signaling assays, we measured the upregulation of a phosphorylated analyte in ITAM-based signaling (p-Syk) in NK cells by receptor engagement using phospho-flow. As with the Luminex platform, we observed an elevation of p-Syk^+^ NK cells stimulated with CD16, whereas NKp46 and CD2 stimulation exhibited only a modest increase in p-Syk^+^ NK cells (medians 0.87%, 55.1%, 4.19%, and 4.69%, unstimulated, CD16-stimulated, NKp46-stimulated, and CD2-stimulated, respectively) (Fig 1C and S1A Fig). Consistent with human NK cells, the phospho-flow analysis demonstrated similar signaling activation profiles in RM NK cells, where CD16 stimulation triggered robust Syk phosphorylation, while NKp46-mediated signaling activation was limited (S1B Fig). Taken together, these findings confirmed that this composite signaling assay was able to monitor the activation of several signaling pathways critical for NK cell activity, and our generated data not only corroborated existing techniques but provided more in-depth characterization of complex NK cell signaling.

### HIV-1/SIV infection specifically suppresses ITAM-based signaling in NK cells

**Fig 2:**
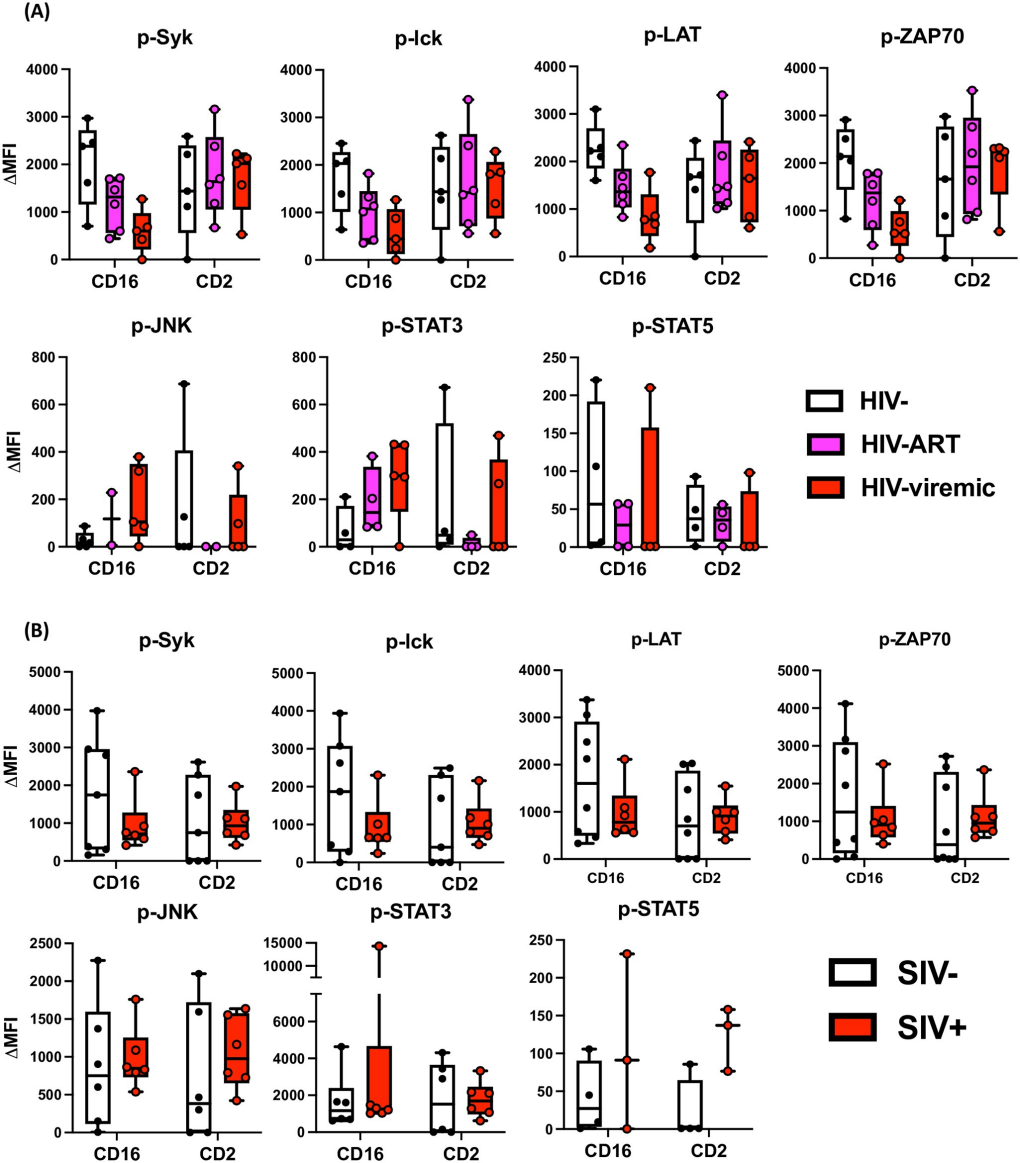
Global ITAM-based signaling perturbation in CD16 signaling pathways during lentiviral infection. **(A)** Human NK cells were isolated from PBMC from healthy control (A; n = 5), PLWH with or without ART (A; HIV-ART, n = 6, HIV-viremic, n = 5, respectively). **(B)** RM NK cell enrichment from SIV-uninfected (n = 8) or SIV-infected (n = 6) animals was also performed. Stimulation via CD16 and CD2 was applied to enriched NK cells, and the levels of the phosphorylated analytes were assessed by Luminex. GAPDH-based normalization of raw MFI and background subtraction using β2M^-^ ‘stimulated’ cells were performed to calculate ΔMFI.

Using the previously described multiplex signaling assay, we next characterized NK cell signaling activation in blood derived from HIV^-^ individuals, PLWH on virally suppressive ART (HIV-ART), and PLWH either off ART or with unsuccessful viremic control (HIV-viremic) (S1 Table). Previous studies have shown that NK cells from PLWH exhibit poorer phosphorylation of ZAP70 following CD16 ligation compared to NK cells from healthy donors [54]. To assess NK cell signaling alterations by HIV-1 infection more broadly, we stimulated NK cells from the blood of healthy donors and PLWH with the same panel of receptors and compared the levels of up to 14 phosphorylated signaling molecules. Our comprehensive signaling analysis revealed that ITAM-based signaling (p-Syk, p-lck, p-LAT, and p-ZAP70) triggered by CD16 stimulation was globally impaired in HIV-viremic individuals compared to HIV-1-uninfected persons (Fig 2A and S3A Fig). Interestingly, ART was not sufficient to fully restore ITAM-based signaling downstream of CD16 (Fig 2A and S3A Fig). Conversely, modest elevations of p-JNK and p-STAT3 were observed in HIV-viremic NK cells following CD16 stimulation, (Fig 2A and S3A Fig). Signaling activation from NKp46, NKG2D, and CD2 crosslinking were not obviously modulated by HIV-1 infection (Fig 2A and S3A and S4A Figs).

Analogous to human studies [53], our previous data showed a reduction in Syk and CD3ζ phosphorylation in NK cells from SIV-infected RM when stimulated with CD16 [42], so we next tested whether SIV-infected RM NK cells also experience a global downregulation of ITAM-based signaling via CD16 crosslinking as seen in PLWH NK cells. Similar to our prior findings, ITAM-based signaling triggered by CD16 crosslinking was broadly dampened in NK cells from SIV-infected RM (Fig 2B and S3B Fig), and a slight activation of STAT5 signaling was observed in NK cells with CD2 stimulation in SIV^+^ animals. Downstream signaling of NKp46 and NKG2D was not significantly modulated by SIV infection (S3B and S4B Figs). In summary, our extensive multiplex signaling analysis provided evidence of broad ITAM-based signaling impairment downstream of CD16 stimulation in both PLWH and SIV-infected RM.

### CD16 downregulation on NK cells during lentiviral infection

**Fig 3:**
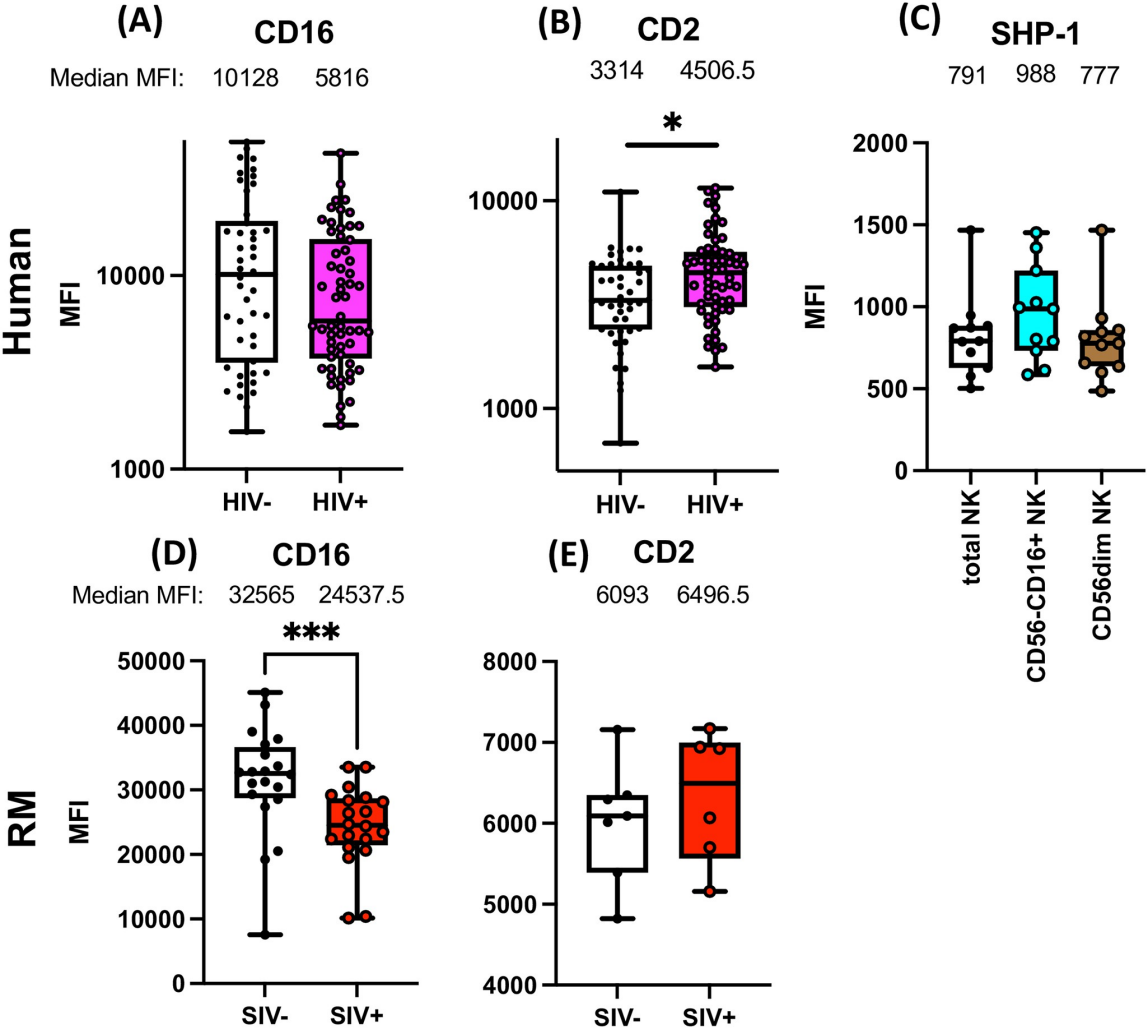
Surface CD16 and CD2 and intracellular SHP-1 expression altered by HIV-1 and SIV infection. **(A, B)** PBMC from HIV-1 uninfected people (A, B; n = 46), and ART-treated (A, B; n = 58) PLWH were stained and the levels of CD16 **(A)** and CD2 **(B)** on live total NK cells were quantified by flow cytometry. **(C)** The levels of SHP-1 on total NK cells, CD56^-^CD16^+^ and CD56dim NK cells from PLWH (n = 11) were measured by flow cytometry. **(D, E)** PBMC from SIV-uninfected or infected (D n = 20, E; n = 6, respectively) RM were stained and the mean fluorescent intensity (MFI) of CD16 **(D)** and CD2 **(E)** was calculated for live CD3^-^CD14^-^CD20^-^HLADR^-^NKG2A/C^+^ cells. Each dot represents different animals or subjects. (*; *p*<0.05, ***; *p*<0.001).

Next, we investigated the potential mechanisms of altered signaling, specifically focusing on CD16 pathways. We first measured the levels of surface CD16 and CD2 expression, as well as intracellular phosphatase SHP-1 expression, which is known to negatively regulate ITAM-based signaling [29]. Multiple groups have reported CD16 downregulation on NK cells in PLWH [21,53,61]. As previously described, NK cells from PLWH included in this study exhibited lower CD16 expression (medians 10128 and 5816, HIV^-^ and HIV^+^, respectively) (Fig 3A and S5A Fig) This CD16 downregulation was not associated with the percentages of CD56^bright^CD16^-^ NK cell subsets in these cohorts (S5B Fig). Conversely, a statistically significant increase in CD2 expression was exhibited by NK cells from PLWH (medians 3314 and 4506.5, HIV^-^ and HIV^+^, respectively) (Fig 3B, *p*<0.05). Others have demonstrated that PLWH accumulate dysfunctional CD56^-^CD16^+^ NK cell populations, unlike a comparative healthy control group [17]. In accordance with their diminished functions, a modest SHP-1 upregulation was specifically exhibited in CD56^-^CD16^+^ NK cells in these individuals (Fig 3C and S5C Fig). The levels of total Syk expression were not significantly altered by HIV-1 infection (S5D Fig).

We hypothesized that SIV infection also modulates CD16 and CD2 expression, thereby altering intracellular signaling in SIV-infected RM. Indeed, a significant reduction of surface CD16 expression was observed in NK cells from SIV^+^ RM (median 32565 and 24537.5, SIV^-^ and SIV^+^, respectively) (Fig 3D and S5E Fig). Additionally, a slight upregulation of CD2 expression was observed on NK cells from SIV^+^ RM, although it was not statistically significant (medians 6093 and 6496.5, SIV^-^ and SIV^+^, respectively) (Fig 3E). Whereas the alteration of surface CD16 and CD2 expression was observed, the expression of total signaling molecules Syk, ZAP70, CD3ζ, and FcRγ was not significantly modulated by SIV infection (S5F Fig). To summarize, we demonstrated that altered CD16 and CD2 signaling in PLWH and SIV-infected RM were associated with lower CD16 and elevated CD2 expression on NK cells.

### IL-18/ADAM17-mediated surface CD16 downregulation during HIV-1 infection

**Fig 4:**
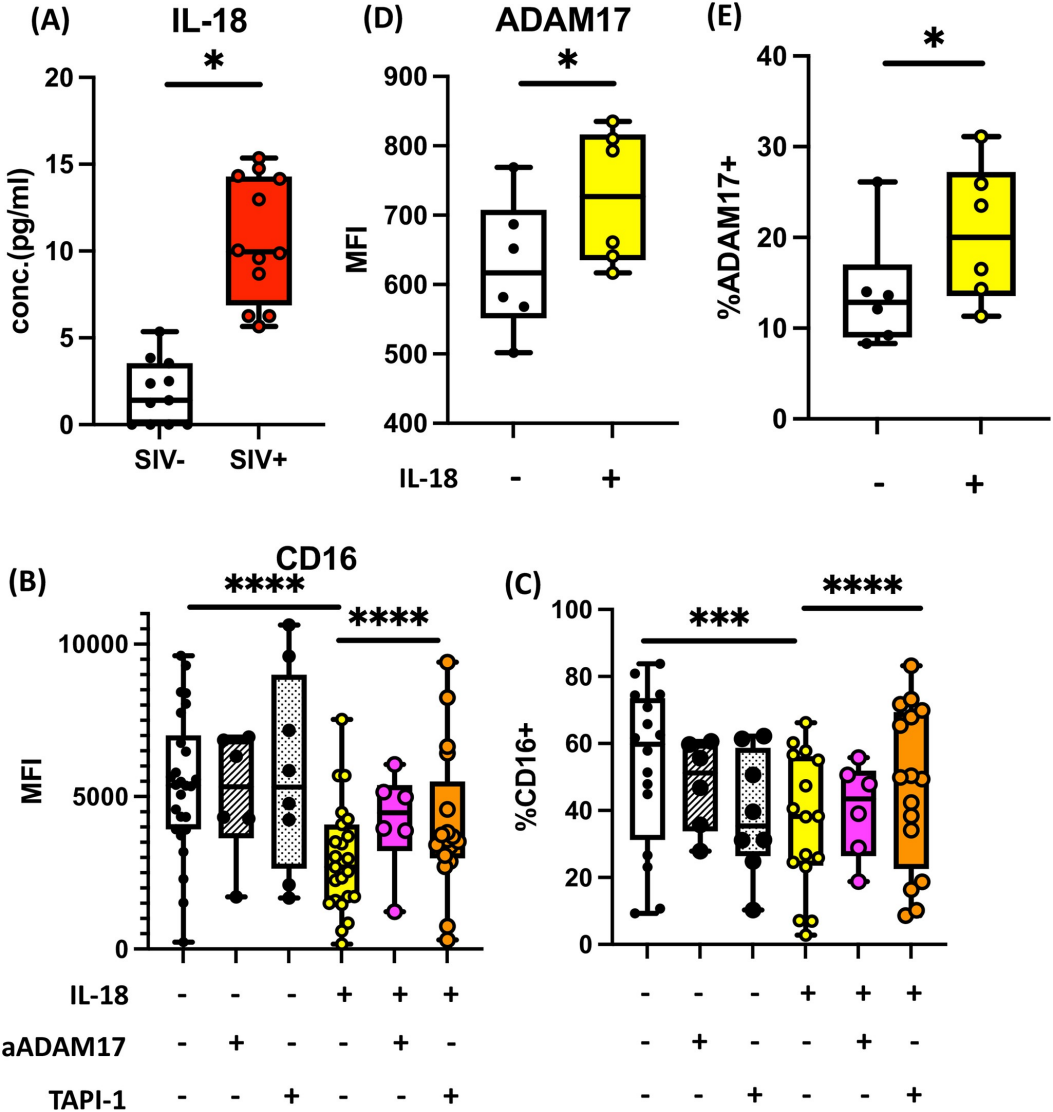
Increased IL-18 in infection elevates ADAM17, systemically diminishing CD16 expression. **(A)** Levels of plasma IL-18 in SIV-uninfected (n = 10), and infected (n = 12) RM were measured by Luminex platform. Shown are summary boxplots with each dot representing an individual animal. **(B, C)** Healthy human PBMC (n = 26) were cultured in the presence of 1ng/mL IL-15 to maintain NK cells viability. Additionally, cells were either left untreated or treated with 10ng/mL IL-18, 6μg/mL ADAM17 blocking antibody (aADAM17), and 25μM TAPI-1. After overnight incubation, the levels of CD16 on NK cells **(B)** and % CD16^+^ NK cells **(C)** gated from PBMC were quantified using flow cytometry. **(D, E)** Healthy human PBMC were treated with or without IL-18 (n = 6) overnight and the levels of ADAM17 on NK cells **(D)** and % ADAM17^+^ NK cells **(E)** were measured by flow cytometry. (*; *p*<0.05, **: *p*<0.005, ***: *p*<0.001).

Next, we examined the mechanisms that may specifically alter surface CD16 and CD2 expression on NK cells during HIV-1/SIV infection. Several studies suggest that NK cell activating cytokines such as IL-12, IL-15, and IL-18 are increased during HIV-1 and SIV infection [62,63]. In our study, we also confirmed that SIV-infected RM showed higher plasma IL-18 levels (medians 1.41pg/ml and 9.96pg/ml, SIV^-^ and SIV^+^, respectively) (Fig 4A). Among these cytokines, it was reported that IL-18 downregulates surface CD16 expression, while IL-12 and IL-15 exhibit limited effects on CD16 modulation [64]. Therefore, we tested whether IL-18 triggers surface CD16 reduction and CD2 upregulation. To do so, we cultured human PBMC in the presence or absence of IL-18, adding low levels of IL-15 to the culture to ensure the optimal viability of the NK cells. Following overnight incubation, we observed that IL-18 reduced surface CD16 expression on NK cells (Fig 4B and 4C). Conversely, CD2 and SHP-1 expression was not altered by IL-15 or IL-18 treatment (S6A Fig). We also investigated whether other IL-1 families (IL-1β and IL-33) can downregulate CD16 expression. IL-33 was not capable of reducing CD16 expression and IL-1β only modestly diminished CD16 expression unlike IL-18 (S6B Fig).

Several studies have implied the roles of a disintegrin and metalloprotease-17 (ADAM17) in CD16 shedding [65], which is suggested to be a potential hurdle for effective NK cell ADCC therapy, including cancer immunotherapy [66,67]. Thus, to identify a putative mechanism of CD16 reduction, we first investigated whether IL-18-mediated CD16 downregulation might be a result of ADAM17 upregulation. We cultured human PBMC in the presence or absence of IL-18 and illustrated that IL-18 stimulation significantly elevated the expression of surface ADAM17 on NK cells (Fig 4D and 4E). Romee et al. demonstrated that decreased CD16 expression can be restored by an ADAM17 inhibitor or blocking antibody against ADAM17 [65]. Another source reported that the small molecule inhibitor TAPI-1 can also perturb ADAM17 functions [68]. Using the ADAM17 blocking antibody and TAPI-1 as described in the literature, we showed that CD16 downmodulation by IL-18 can be reversed by blocking ADAM17 functions (Fig 4B and 4C). We also confirmed that RM IL-18 triggers CD16 reduction dependent on ADAM17 activity (S7 Fig). Thus, we propose that HIV-1/SIV infection elevates plasma IL-18 levels, which increases NK cell ADAM17 expression and thereby diminishes surface CD16 expression.

### IL-18/ADAM17 suppresses CD16 signaling activation

**Fig 5:**
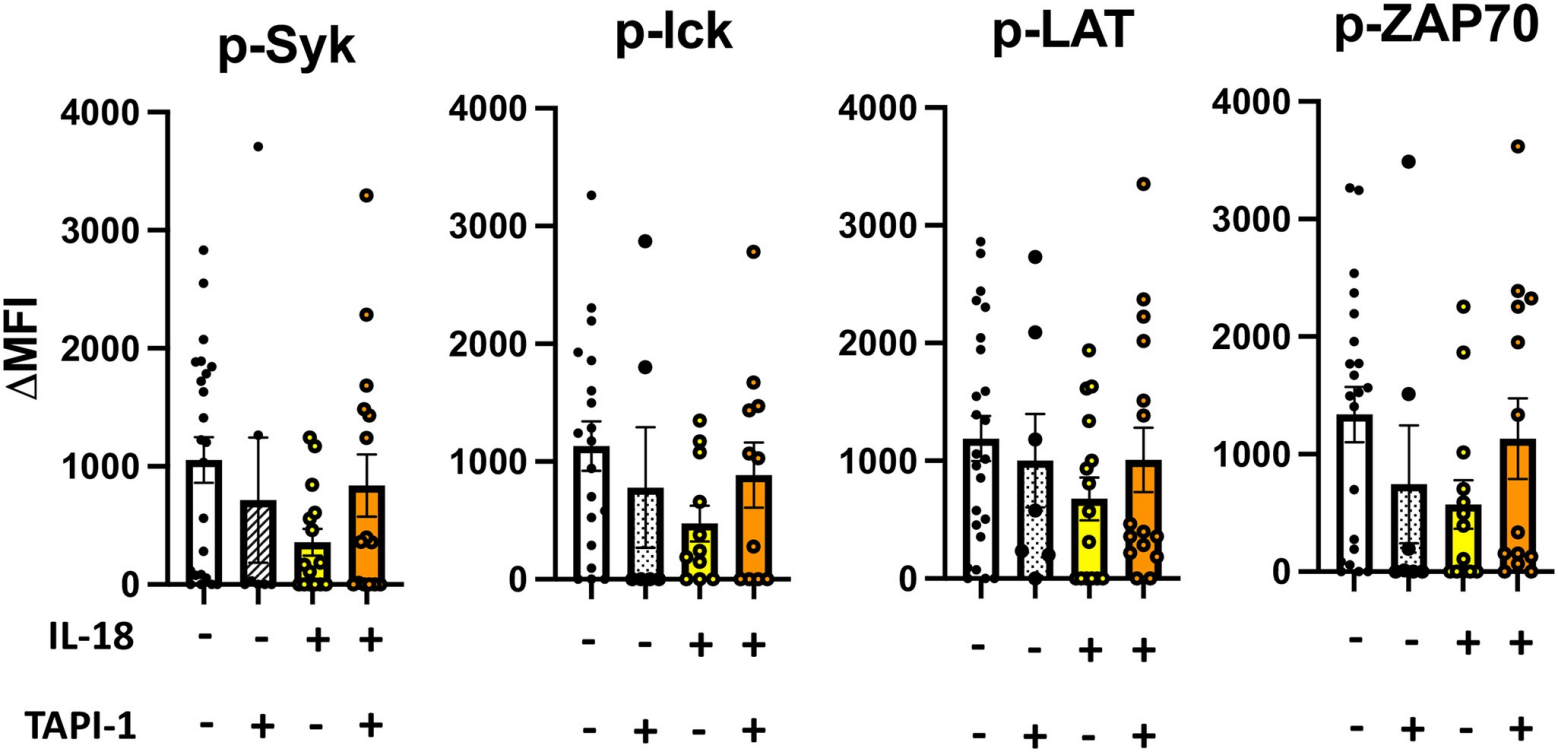
IL-18/ADAM17 pathway systemically diminishes CD16 signaling activation in NK cells. Healthy human PBMC (n = 21) were left untreated or incubated with or without 10ng/mL IL-18, and 25μM TAPI-1. For all conditions, 1ng/mL IL-15 was added to maintain NK cell viability. NK cells were then isolated and stimulated with anti-CD16 antibody. Total protein was harvested and levels of p-Syk, p-lck, p-LAT, and p-ZAP70 were measured by Luminex signaling assay. Summary boxplots of phosphorylated protein levels are shown. Each subject is shown as a separate dot. The raw MFI of each analyte was normalized by loading control GAPDH and ΔMFI was calculated by subtracting normalized MFI from β2M-stimulated cells. (*: *p*<0.05, **: *p*<0.01).

We next investigated whether reduced CD16 expression induced by IL-18/ADAM17 affects ITAM-based signaling activation in the CD16 pathway. Corresponding with diminished CD16 expression, IL-18 dampened ITAM-based signaling activation by CD16 stimulation in NK cells (Fig 5 and S8 Fig). Subsequently, we tested whether reduced CD16 signaling by IL-18 can be restored by inhibiting ADAM17 functions. Through recovering CD16 expression (Fig 4B and 4C), ADAM17 inhibition by TAPI-1 also restored ITAM-based signaling by CD16 stimulation in NK cells (Fig 5 and S8A Fig). The magnitude of Syk phosphorylation was also positively correlated with the CD16 expression in this experiment, further supporting the finding that impairment of CD16 signaling is dependent on IL-18/ADAM17-mediated surface CD16 regulation (S8B Fig). Taken together, we demonstrated that the downregulation of NK cell surface CD16 expression by an IL-18/ADAM17-dependent mechanism results in reduced downstream intracellular signaling.

### ADAM17 inhibition restores CD16 expression in NK cells from PLWH

**Fig 6:**
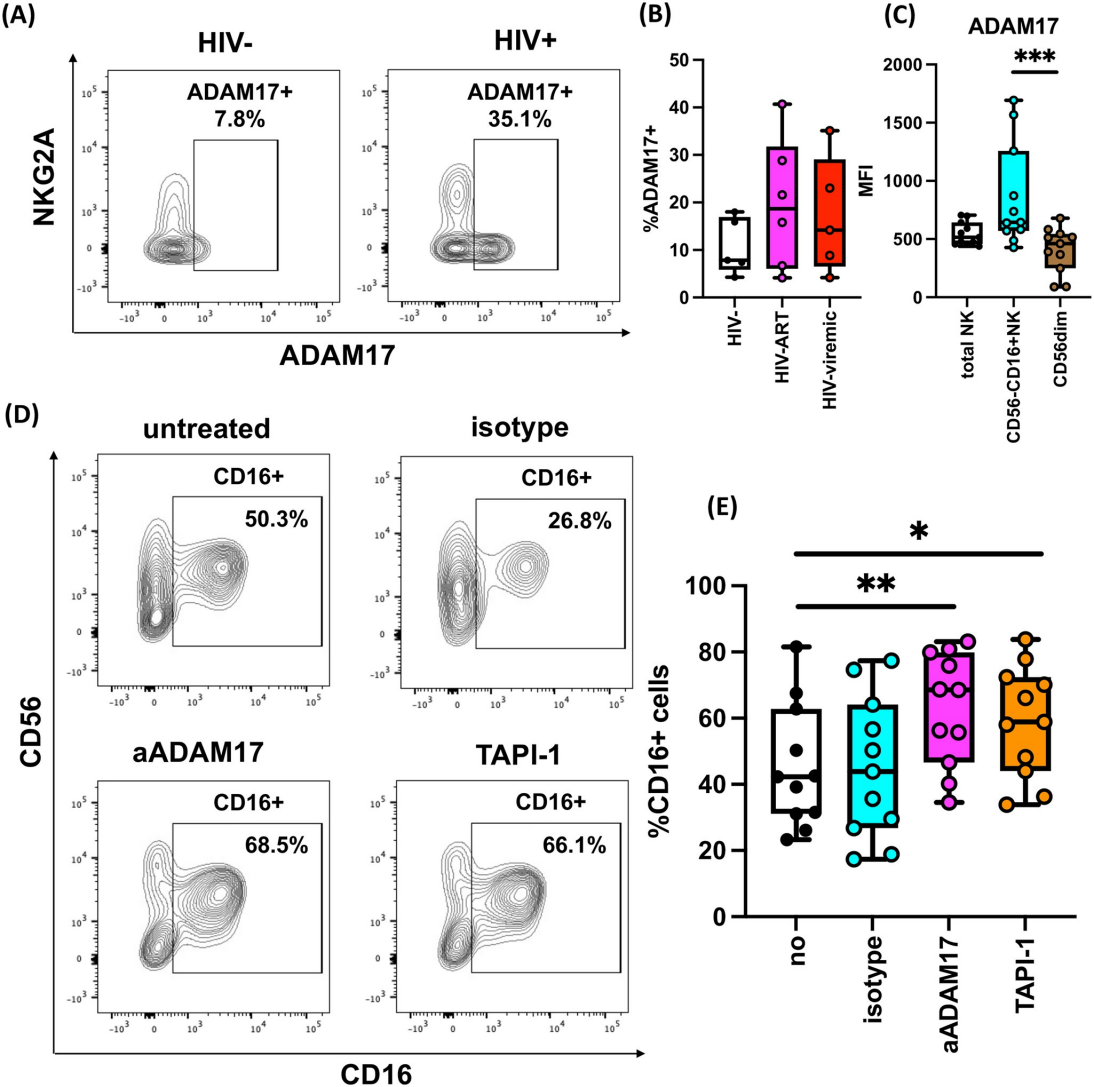
PLWH exhibited upregulated ADAM17 expression mediating CD16 downregulation. **(A, B, C)** PBMC from HIV-1-uninfected people (n = 5), and PLWH with (n = 6) or without ART (n = 5) were thawed and stained, and the percentages of ADAM17^+^ NK cells were calculated. **(A)** Representative flow plots from NK cells in healthy donors and PLWH are shown. **(B)** Summary boxplots for %ADAM17^+^ NK cells for total NK cells among groups were shown. **(C)** %ADAM17^+^ NK cells for total, CD56^-^CD16^+^ and CD56dim NK cells from PLWH (n = 11) were summarized in boxplot. **(D, E)** PBMC from PLWH with or without ART were cultured in the presence or absence of human IgG1 isotype, ADAM17-blocking antibody (aADAM17), or TAPI-1 (n = 11, respectively). Following overnight incubation, the percentages of CD16^+^ cells from live CD3^-^CD14^-^CD20^-^ cells were calculated by flow cytometry. **(D)** Representative flow plots for live CD3^-^CD14^-^CD20^-^ cells were depicted. **(E)** Shown were the summary boxplots for %CD16^+^ cells from CD3^-^CD14^-^CD20^-^ cells where individual dots depict each subject (n = 5). (*; *p*<0.05, **; *p*<0.01).

To validate our findings *ex vivo*, we investigated the role of ADAM17 on systemic downregulation of CD16 signaling in blood specimens derived from HIV-ART and HIV-viremic groups. We demonstrated that total NK cells from PLWH had higher percentages of ADAM17^+^ NK cells compared to HIV^-^ donors, regardless of ART status (Fig 6A and 6B). When we assessed CD56^-^CD16^+^ NK cells specifically, HIV-1-induced upregulation of ADAM17 became even more evident (medians 516, 644, and 461, total NK cells, CD56^-^CD16^+^, and CD56^dim^ NK cells respectively, *p*<0.005) (Fig 6C). This indicates that defective CD56^-^CD16^+^ NK cells, a distinct NK cell subset during HIV-1 infection, preferentially increase ADAM17.

Finally, we tested whether the reduced CD16 expression observed on NK cells from PLWH can be recovered via blocking of ADAM17 function. As expected, we demonstrated that administration of an ADAM17 blocking antibody or ADAM17 inhibitor TAPI-1 increased the percentages of CD16^+^ NK cells in PLWH (medians 42.3%, 43.9%, 68.5%, and 58.9%, untreated, isotype, aADAM17, and TAPI-1 respectively, *p*<0.05) (Fig 6D and 6E). Taken together, our findings illustrate that this subset of NK cells in PLWH also increases ADAM17 expression, which results in lower CD16 expression and subsequent CD16 signaling. Further, we also observed that diminished CD16 expression on NK cells in PLWH can be recovered by inhibiting ADAM17.

## Discussion

Herein, we have established a multiplex NK cell signaling assay with the capability to extensively characterize both human & RM NK cell signaling activation. Using this assay, we elucidated that ITAM-based signaling in the CD16 pathway was globally downregulated in PLWH and SIV-infected RM. Intriguingly, JNK and STAT3 activation by CD16 stimulation was modestly enhanced NK cells derived from PLWH. For SIV-infected RM, increased STAT5 activation upon CD2 stimulation was illustrated in addition to modulated CD16 signaling. We further demonstrated that altered signaling profiles were directly related to surface CD16 and CD2 expression profiles while moderate SHP-1 upregulation was observed specifically in CD56^-^CD16^+^ NK cells from PLWH. To explore the potential mechanism, we developed an *in vitro* system to assess CD16 downregulation, and we found that IL-18 significantly decreased NK cell CD16 expression and subsequent ITAM-based signaling activation. Additionally, we found these changes to be dependent on IL-18-induced ADAM17, which cleaves the surface domain of CD16 [65], and that IL-18-mediated CD16 reduction can be reversed via ADAM17 blockade. Finally, we observed an elevated ADAM17 expression in NK cells in PLWH, and that CD16 levels can be recovered by inhibition of ADAM17 function.

Our study highlights several critical findings for NK cell biology. First, our multiplex signaling assay was able to provide evidence for a more thorough intracellular NK cell signaling profile during lentiviral infection than has ever been characterized. While the diminished phosphorylation of CD3ζ, Syk, and ZAP70 by CD16 stimulation has been reported in NK cells from PLWH and SIV-infected RM [42,53], these studies were able to monitor only a few molecules per experiment due to the technical limitations of using Western Blotting or phospho-flow [29]. Since these techniques detect only limited numbers of analytes simultaneously, prior studies could not differentiate whether HIV-1/SIV infection specifically perturbs distinct kinases in the cascades or systemically downregulates signaling pathways. To our knowledge, a more complete NK cell signalome profile during HIV-1/SIV infection has never been characterized. Our multiplex NK cell signaling assay revealed a broad downregulation of ITAM-based signaling in CD16 cascades within NK cells during lentivirus infection, more rigorously described than previous studies [42,53]. In addition, our multiplex signaling analyses also revealed that JNK and STAT3 phosphorylation increased within NK cells from PLWH after CD16 stimulation. The increase in JNK and STAT3 phosphorylation in the CD16 pathway may imply the global activation of inflammatory pathways independent of CD16 crosslinking. Moreover, because these molecules are less proximal to the receptors, it would be difficult to interpret their activation in the context of short receptor crosslinking. Thus, ITAM-based signaling can serve as a more specific readout from CD16 signals compared to downstream molecules, including JNK and STAT3. Nevertheless, because IL-18 also induces the STAT3 pathway [70], increased p-STAT3 levels further validate that IL-18 signaling is involved in the systemic impairment of CD16 signaling as we hypothesized. Altogether, our novel multiplex signaling analysis demonstrated that lentiviral infection globally diminishes ITAM-based signaling, not specifically targeting molecules, downstream of CD16 engagement.

Secondly, our study demonstrated that human and RM NK cells exhibit similar signaling activation profiles upon receptor crosslinking. Several groups reported that RM NK cells rigorously recapitulate human NK cell responses [70,71], but their signaling activation profiles have yet to be explored. Our results imply that analogous signaling activation profiles were observed in NK cells from both humans and RM. It is important to note that there is disparate signaling activation by NKp46 and CD2 stimulation between human and RM NK cells. These differences might be attributed to less optimal binding to RM NKp46 and CD2 by anti-human NKp46 and CD2 antibodies.

In addition to the comparable signaling activation between human and RM NK cells, our study revealed that downregulated CD16 expression coincides with reduced downstream signaling pathway activation. Many groups have comprehensively characterized dysfunctional NK cell responses associated with altered NK cell surface receptor expression [14,16–20,24,28], but surface receptor alteration does not always predict NK cell activities. Indeed, Nielsen et al. reported that, while IL-18 treatment on NK cells reduced CD16 expression, NK cell ADCC was enhanced by the addition of IL-18, presumably due to increased NK cell activation [64]. Therefore, it is unclear how the perturbation of NK cell receptor expression by lentiviruses predicts intracellular signaling activation status and subsequent NK cell effector responses. This study was able to provide the missing link between surface receptor profiles and downstream intracellular signaling activation and confirm that NK cell surface receptor expression profiles during HIV-1/SIV infection do infer their signaling profile as well as their functionality.

Along with the link between surface receptor expression and downstream intracellular signaling, this study explicated the reduction of CD16 via an IL-18/ADAM17-driven mechanism in HIV-1/SIV infection, as seen in other viral infections. Individuals with herpes simplex virus infection have been reported to exhibit reduced CD16 expression on NK cells [72]. Diminished CD16 expression on NK cells has also been characterized in individuals with chronic hepatitis C virus (HCV) infection, and this is partly triggered by elevation of ADAM17 expression [73]. Olivero et al. also demonstrated that the clearance of HCV by direct-acting antivirals partially restores CD16 expression on NK cells and subsequent ADCC activity [73]. This indicates that CD16 downregulation could be reversible by therapeutic treatments. Our study illustrated that, analogous to other viral infections, HIV-1/SIV infection also reduced CD16 expression via ADAM17 elevation, and this interaction can be therapeutically targeted to restore NK cell functions. Intriguingly, PLWH receiving ART maintained ADAM17 expression while their responsiveness to CD16 crosslinking was diminished. This discrepancy may have derived from an increase in defective CD56^-^CD16^+^ NK cell populations [16–20] from PLWH on ART. Although our data also indicated that SHP-1 expression was specifically elevated in this subset, this was somewhat inconclusive due to infrequent events for some of the assays. Since this population systemically experiences the downregulation of overall NK cell functions [16–20], an IL-18/ADAM17-dependent pathway alone cannot fully delineate the mechanisms of impaired CD16 signaling in total NK cells from PLWH.

While PLWH NK cells decreased CD16 expression, they significantly elevated CD2 expression. This could result from accumulation of adaptive NK cell subsets in PLWH, which are characterized by increased CD2 expression [44,74]. Conversely, in SIV-infected RM, only moderate increase in CD2 levels was observed on NK cells. It was plausible that CD2 upregulation was modest in this study due to the limited sample size of SIV-infected animals. If we could increase the number of SIV^+^ animals in this experiment, CD2 elevation would be expected to become more apparent on NK cells during SIV infection similar to PLWH NK cells.

Our findings indicate that ADAM17 could be considered a promising target for NK cell HIV-1 immunotherapies similar to cancer immunotherapies. ADAM17-mediated CD16 reduction has been increasingly studied for NK cell-based cancer immunotherapy as a strategy to optimize NK cell ADCC [75]. While the targeting of lymphomas with the ADCC-inducing antibody Rituximab does trigger NK cell activation [76], CD16 expression was diminished on CD56^dim^CD16^+^ NK cells in Rituximab recipients 12 months after treatment [66]. To overcome this problem, Pomeroy et al. established ADAM17-deficient primary human NK cells by CRISPR and demonstrated that CD16 expression was restored and their ADCC activities against tumors were ameliorated [67]. Furthermore, NK cells become resistant to ADAM17-mediated CD16 cleavage by introducing mutations in ADAM17-cleavage site [77]. While these strategies seem promising for HIV-1 treatment and cure, adoptive NK cell therapy is the only NK cell-based immunotherapy tested for PLWH in clinical trials thus far, unlike cancer therapies where application of NK cells has been established [29]. However, our findings on ADAM17-mediated CD16 reduction in PLWH indicate that techniques in NK cell cancer immunotherapy such as ADAM17 CRISPR knockout and ADAM17-resistant CD16 overexpression could also be translated for use as NK cell-based HIV-1 immunotherapeutics. Therefore, this study has revealed the potential translation of NK cell cancer immunotherapy techniques into HIV-1 immunotherapeutics. Additionally, ADAM17 is known to regulate expression of diverse cytokine receptors, cell adhesion molecules, and homing receptors [78]. Given the possibility of ADAM17 altering multiple NK cell activities [78], ADAM17-based therapies can also enhance NK cell homing and improve responses to cytokines for better HIV-1 treatment.

In conclusion, our multiplex NK cell signaling assay delineated distinct CD16 signaling downregulation during HIV-1/SIV infection, which corresponded with surface CD16 and CD2 expression. We also elucidated that ADAM17 upregulation by IL-18 reduces NK cell surface CD16 expression and subsequent intracellular signaling activation. These findings will inform future NK cell-based HIV-1 immunotherapeutics aimed at both restoring and enhancing NK cell activity against HIV-1.

## Methods

### Ethics statement

All Indian-origin RM (*Macaca mulatta*) were housed at Biomere, Inc., Worcester, MA in compliance with the rules and regulations of the Committee on the Care and Use of Laboratory Animal Resources. The housing of animals was performed following the American Association for Accreditation of Laboratory Animal Care standards and *The Guide for the Care and Use of Laboratory Animals*. All animal experiments were reviewed and approved by the Biomere Institutional Animal Care and Use Committee (IACUC) under protocols #16–08, 17–02, and 20–02, and in agreement with ARRIVE (Animal Research: Reporting of In Vivo Experiments) guidelines. Standard monkey chow diet supplemented daily with fruit and vegetables and water *ad libitum* was supplied for animals tested. Veterinary staff provided social enrichment and monitored overall animal health. Clinically significant signs of weight loss, disease, or distress were assessed for each animal, and dietary supplementation, analgesics, and/or therapeutics were supplied if necessary. RM were humanely euthanized by an overdose of barbiturates as recommended by the guidelines of the American Veterinary Medical Association. IACUC protocols 20–02, 17–02, and 16–08 were reviewed and approved by the Biomere standing IACUC. If needed, immobilization of animals was performed by intramuscularly injecting ketamine HCl (Parke-Davis) at approximately 10mg/kg following overnight fasting. Venipuncture was used to harvest blood samples.

### Animal samples

Blood samples from 28 RM (23 male and 5 female animals) were used for these experiments. The animals were infected with SIVmac251, and blood collection was performed prior to SIV infection and at the chronic stage of infection when sacrificing animals [79]. PBMC were purified from whole blood by Ficoll-Hypaque-based density gradient centrifugation followed by lysis of red blood cells using hypotonic ammonium chloride solution.

### Human samples

Healthy human blood was sampled under protocols approved by Research Blood Components Institutional Review Board (Watertown, Massachusetts) and Zen-Bio Inc. Institutional Review Board (Durham, North Carolina). PBMC were separated by Ficoll-Hypaque-based density gradient centrifugation. Cohorts of additional HIV-1-uninfected subjects and PLWH were established and approved by University of Alabama at Birmingham Hospital Institutional Review Board, Duke University Hospital Institutional Review Board, and University of Hawaii Hospital Institutional Review Board [6]. All participants signed IRB-accredited informed-consent form prior to this study. The demographic characteristics of each cohort are summarized in S1 Table.

### NK cell enrichment and Luminex analysis

Human NK cells were isolated from PBMC by EasySep Human NK cell isolation kit (STEMCELL technology) or Human NK cell Isolation kit (Miltenyi) and AutoMACS Separator (Miltenyi) following the manufacturer’s recommended protocols. NK cells from RM PBMC or spleens were enriched by the previously described protocol [80,81].

Harvested NK cells were incubated with 200ng/ml anti-human CD16, (BD Biosciences, clone 3G8), 20μg/ml anti-human NKp46 (Beckman Coulter, clone BAB281), 20μg/ml anti-human NKG2D (R&D systems, clone MAB139), or 2μg/ml anti-human CD2 antibody (BD Bioscience, clone RPA-2.10). 20 minutes after incubation, goat anti-mouse IgG F(ab)’2 fragments (GAM; 40μg/ml, Jackson ImmnoResearch Laboratory) were added to crosslink antibodies for 5 minutes at 37°C. The doses of each stimulant and the length of crosslinking were determined by antibody titration and time course experiments and also referenced from our previously published study [42]. Since the crosslinking reagent GAM alone could trigger some ITAM-based, STAT and MAPK signaling, we prepared replicates with control 20μg/ml anti-human β2-microbulin (β2M; R&D systems, clone 2M2) to account for the background signals from GAM in the presence of an antibody. Matched samples were then used as background controls. Stimulated cells were then lysed using Milliplex lysis buffer (EMD Millipore) supplemented with protease inhibitor cocktail set III (EMD Millipore) and total protein was collected.

### Luminex signaling analysis

The levels of 10 phosphorylated (p) proteins (p-Syk, p-lck, p-LAT, p-ZAP70, p-JNK, p-NFκB, p-p70S6K, p-Akt, p-STAT3, and p-STAT5) in NK cell lysates were quantified using Milliplex Multi Pathway Magnetic Bead 9-Plex (EMD Millipore) and Milliplex T cell receptor signaling Magnetic Bead kit 7-plex (EMD Millipore) following manufacturer’s recommended protocols and data was collected on the MAGPIX System and Luminex 200 system (Luminex Corp). The raw mean fluorescent intensities (MFI) of phosphorylated proteins were normalized by the amount of GAPDH in each sample measured by Milliplex MAP Total GAPDH Magnetic Bead MAPMATE (EMD Millipore). ΔMFI was calculated using cells stimulated with anti-β2M antibody for background subtraction. Signals lower than β2M-‘stimulated’ conditions were normalized to an MFI of one. We also separately analyzed GAPDH-normalized MFI values without background subtraction for each experiment, which is illustrated in S3 and S8 Figs.

### Luminex cytokine analysis

Plasma IL-18 levels in the plasma of SIV-uninfected or -infected RM were measured using IL-18 Non-Human Primate Procarta Simplex kit (ThermoFisher) following manufacturer’s recommended instructions. Luminex 200 system (Luminex Corp) was used for data acquisition, and the concentration of IL-18 in each sample was calculated by ProcartaPlex Analyzer App (ThermoFisher) based on the standard curve generated from the same experiment. Undetectable cytokine concentrations were normalized to 0.01pg/ml, the limit of detection for this assay.

### Flow cytometry

Mononuclear cells and PBMC were stained with viability dye Aqua 500 solution (ThermoFisher) for 30 minutes followed by the staining of surface receptors: anti-CD3-BV786 (BD Pharmingen, clone SP34.2), CD14-BUV737 (BD Pharmingen, clone M5E2), CD20-BUV395 (BD Pharmingen, clone L27), CD56-BV605 (BD Pharmingen, clone NCAM16.2), HLADR-APC-Cy7 (BD Pharmingen, clone G46-6), CD16-BUV496 (BD Pharmingen, clone 3G8), NKp46-PE-Cy5.5 (Beckman Coulter, clone BAB281), NKG2D-APC (Miltenyi Biotec, clone BAT221), CD2-PE-CF594 (BD Pharmingen, clone RPA2.10), anti-NKG2A-PE-Cy7 (Beckman Coulter, clone Z199), and anti-ADAM17-Pacific Blue. Anti-ADAM17-Pacific Blue antibodies were generated by conjugating fluorophore to anti-human ADAM17 antibody (R&D systems, clone MAB9304) using the Pacific Blue conjugation kit (ThermoFisher). 20 minutes after surface receptor staining, cells were permeabilized with the Fix & Perm buffer kit (ThermoFisher) and stained with an intracellular staining antibody cocktail including anti-Syk-PE (Life Technologies), anti-Fc receptor γ chain (FcRγ)–Alexa Fluor 700 (conjugated in our lab from Anti-FcεRI antibody γ subunit, EMD Millipore, rabbit polyclonal), and anti-SHP-1-FITC (G-Bioscience, rabbit polyclonal) for 20 minutes. Cells were then fixed with 2% paraformaldehyde and data were collected by LSR II and A5 Symphony (BD Biosciences), and FlowJo v.9.9.6 (Flowjo) was used for analysis. The gating strategies for NK cells in human and RM PBMC were summarized in S2A and S2B Fig respectively and isotype or FMO controls were employed where applicable. We also adopted several mitigation approaches to minimize the potential batch effects during our experiments. First, we purchased the antibodies in bulk so that we used a single lot of antibodies for the entire duration of this project. Secondly, quality control of the BD FACS Symphony, including the use of calibration beads [82], was performed daily to ensure that the voltages, filters, and lasers were within consistent and acceptable values. Finally, the status of the samples was not disclosed for each analysis day, and each sample group was consequently sufficiently mixed.

### Phospho-flow

Human or RM PBMC were thawed and incubated with 200ng/ml of anti-CD16 (BD Bioscience, clone 3G8), 20μg/ml of anti-NKp46 antibody (Beckman Coulter, clone BAB281), or 2μg/ml anti-human CD2 antibody (BD Bioscience, clone RPA-2.10) at room temperature. 20 minutes after incubation, antibodies on cells were crosslinked with 40μg/ml of GAM for 2.5 minutes at 37°C. PBMC were then fixed with BD fixation buffer I (BD Biosciences) for 10 minutes at 37°C and blocked with normal mouse serum (ThermoFisher) for 10 minutes. After blocking, surface receptors were stained using anti-CD3-BV786 (BD Pharmingen, clone SP34.2), anti-CD14-BUV737 (BD Pharmingen, clone M5E2), anti-CD20-BUV395 (BD Pharmingen, clone L27), anti-CD56-BV605 BD Pharmingen, clone NCAM16.2), anti-HLADR-ECD (Beckman Coulter, clone IMMU-357) for 20 minutes at room temperature. Stained cells were then permeabilized by BD Perm buffer III (BD Biosciences) for 30 minutes on ice and stained with anti-CD3ζ-PE-Cy5.5 (Novus Biological, clone H146-968), anti-phospho (p)-CD3ζ-APC (BD Pharmingen, clone K25.-407.69), anti-Syk-PE (Life Technologies, clone 4D10.1), anti-p-Syk-AlexaFluor 488 (Cell Signaling, rabbit polyclonal), and FcRγ- AlexaFluor 700 (EMD Millipore, rabbit polyclonal, conjugated in our lab). 20 minutes after staining, cells were fixed with 2% paraformaldehyde. Data were acquired by LSR II and A5 Symphony (BD Biosciences), and the analysis was performed using FlowJo v.9.9.6.

### Cytokine and anti-ADAM17 blocking antibody effects on NK cell surface receptor expression and signaling

Human PBMC were cultured in RPMI Medium (Corning) with 10% Fetal Bovine Serum (ThermoFisher) (R10) in the presence or absence of 1ng/ml recombinant human IL-15 (Miltenyi Biotec), 10ng/ml recombinant human IL-18 (R&D systems), 6μg/ml human IgG1 kappa isotype control (Abcam, clone MOPC-21), 6μg/ml anti-TACE antibody (EMD Millipore, clone D1[A12]), and 25μM ADAM17 inhibitor TAPI-1 (Selleckchem) [65,68] overnight. The doses of cytokines and inhibitors, and length of incubation were determined based on previously published studies [65,68]. PBMC were then stained with anti-CD3-BV786, CD14-BUV737, CD20-BUV395, CD56-BV605, ADAM17-Pacific Blue, HLADR-APC-Cy7, CD16-BUV496, NKp46-PE-Cy5, CD2-PE-CF594, and anti-NKG2A-PE-Cy7. Following surface receptor staining, cells were permeabilized with the Fix & Perm buffer kit and stained for intracellular markers (anti-Syk-PE, FcRγ-AlexaFluor700, and SHP-1-FITC). Stained cells were then analyzed by flow cytometry.

In parallel, NK cells were isolated from PBMC after overnight culture using human NK cell isolation kit (Miltenyi) and AutoMACS Separator (Miltenyi). Cells were coated with 20μg/ml anti-human β2-microbulin (β2M; Biolegend, clone 2M2), 200ng/ml anti-human CD16 (BD Biosciences, clone 3G8) for 20 minutes followed by antibody crosslinking using 40μg/ml GAM for 5 minutes at 37°C. Cells were then lysed and the levels of phosphorylated molecules in ITAM-based signaling (p-Syk, p-lck, p-LAT, and p-ZAP70) were quantified by the Luminex 200 system (Luminex Corp) using Milliplex T cell receptor signaling Magnetic Bead kit 7-plex (EMD Millipore) and Milliplex MAP Total GAPDH Magnetic Bead MAPMATE (EMD Millipore).

For RM PBMC, cells were incubated in R10 with or without 10ng/ml recombinant RM IL-18 (R&D systems), 6μg/ml anti-TACE antibody (EMD Millipore, clone D1[A12]), and 25μM ADAM17 inhibitor TAPI-1 (Selleckchem). The next day, cells were stained with the same surface & intracellular antibody panel as with human PBMC, and the levels of CD16 on NK cells were measured via flow cytometry.

### Statistical analysis

Statistical analyses were performed using GraphPad Prism 9.0 software (GraphPad software). *P*-values of less than 0.05 were considered to be statistically significant. For unpaired samples, One-way ANOVA tests were used to determine statistically significant differences in experiments with more than three conditions. For experiments between two groups, Mann-Whitney *U* tests were applied to evaluate statistically significant differences for unpaired analysis, and Wilcoxon matched-paired signed rank tests or paired *t* tests were performed for paired analyses. When analyzing the correlation between two parameters, Spearman correlation coefficient was calculated using GraphPad Prism 9.0 software.

## Supporting information

S1 Table Cohort demographics. Demographic information on HIV-uninfected individuals and PLWH with or without antiretroviral treatments (HIV-ART & HIV-viremic, respectively) were tabulated. VL: viral load, NA: not applicable.
(XLSX)

S1 Fig Phospho-flow analysis on human and RM NK cells stimulated with activating receptors. Healthy human PBMC **(A)** and experimentally naïve RM PBMC **(B)** were crosslinked with anti-CD16 or NKp46 antibody and the percentages of p-Syk^+^ cells in live CD3^-^CD14^-^CD20^-^CD56^+^Syk^+^ were quantified. Representative flow plots are shown.
(TIF)

S2 Fig The gating strategy for phenotyping human and RM PBMCs. Shown are representative gating strategies to analyze surface and intracellular marker expression on HIV-1-uninfected or PLWH human PBMC **(A)** and RM PBMC with or without SIV infection **(B)**.
(TIF)

S3 Fig Normalized MFI values of phospho-proteins after stimulation of NK cells from PLWH and SIV+ RM prior to background subtraction. **(A)** NK cell isolation was performed for PBMC from HIV-1 uninfected individuals (n = 5), PLWH on (n = 6) or off ART (n = 5) and crosslinked with anti-CD16, NKp46, NKG2D, and CD2 antibodies. The levels of phosphorylated proteins were measured by Luminex technology. MFI values of each analyte were normalized by GAPDH MFI, and summary boxplots were shown. **(B)** NK cells were enriched from naïve (n = 8) and SIV chronically-infected RM (n = 6) and stimulated via CD16, NKp46, NKG2D, and CD2 crosslinking. The amount of phosphorylated analytes was quantified by Luminex platform. normalized MFI by GAPDH levels were plotted in the summary boxplots.
(TIF)

S4 Fig Signaling activation profiles in NK cells stimulated with NKp46 and NKG2D during HIV-1/SIV infection. **(A)** NK cells were purified from PBMC from HIV-1 uninfected subjects (n = 5), PLWH with (n = 6) or without ART (n = 5) and stimulated with NKp46 and NKG2D. the levels of phospho-proteins were measured by Luminex platform. **(B)** PBMC from SIV-uninfected (n = 8) and chronically infected (n = 2) were enriched for NK cells. Cells were then crosslinked with anti-NKp46 and anti-NKG2D antibodies and the magnitude of phosphorylation of seven signaling molecules was assessed by Luminex assay. ΔMFI was calculated by normalization of raw MFI by GAPDH values followed by background subtraction using β2M- ‘stimulated’ cells. The summary boxplots are depicted, and each dot represents different donors or animals.
(TIF)

S5 Fig Frequency of CD16^+^ NK cells and signaling molecule expression in NK cells during chronic lentiviral infection. **(A)** PBMC from HIV-1 uninfected individuals (n = 46), and PLWH receiving ART (n = 58) were stained and %CD56dimCD16^+^ NK cells of live CD3^-^CD4^-^CD14^-^CD19^-^ were calculated by flow cytometry. **(B)** The percentages of CD56brightCD16- NK cells were correlated with the expression of CD16 on total NK cells. Spearman correlation coefficient was calculated. **(C, D)** The levels of SHP-1 **(C)** and total Syk **(D)** in total NK cells from HIV-1-uninfected subjects (n = 5), PLWH with (n = 6) or without ART (n = 5) were measured by flow cytometry. **(E)** PBMC from SIV-uninfected or infected (n = 20) RM were stained and %CD56^-^CD16^+^ cells was measured for live CD3^-^CD14^-^CD20^-^HLADR^-^NKG2A/C^+^ cells. Each dot represents different animals or subjects. (***: *p*<0.005). **(F)** The expression of total Syk, ZAP70, CD3ζ, and FcRγ in total NK cells from SIV-uninfected (n = 7), and chronically infected (n = 6) macaques were quantified by flow cytometry.
(TIF)

S6 Fig The effect of IL-15 and IL-1 family cytokines on surface CD2, CD16, and intracellular SHP-1 expression on NK cells. **(A)** Healthy human PBMC were left untreated (n = 12) or incubated with IL-18 (n = 10), IL-15 (n = 6), or both IL-15 & IL-18 (n = 6). After overnight culture, cells were then stained and analyzed by flow cytometry. The MFI of CD2 and SHP-1 on live total NK cells were quantified. Summary boxplots of CD2 and SHP-1 MFI on live total NK cells were shown, where respective dots represent different subjects. **(B)** Healthy human PBMC were cultured in the presence or absence of IL-1β, IL-33, and IL-18 overnight and the expression of CD16 on total NK cells were measured by flow cytometry. The statistically significant differences were evaluated using paired *t* tests. (n = 4, *: *p*<0.05).
(TIF)

S7 Fig RM IL-18 diminishes NK cell CD16 expression in an ADAM17-dependent fashion. SIV-uninfected RM PBMC were incubated with or without IL-18, ADAM17 blocking antibody (aADAM17), and TAPI-1 (n = 2 for each). Following incubation, cells were stained and flow cytometric analysis was performed to assess the levels of CD16 on NK cells.
(TIF)

S8 Fig Normalized MFI values of phospho-proteins after stimulation of NK cells treated with IL-18 and TAPI-1 without background subtraction. **(A)** Healthy human PBMC were cultured in the presence or absence of 10ng/mL IL-18 (n = 8) and 25μM TAPI-1 (n = 8) along with IL-15 overnight. NK cells were then isolated and then applied with CD16 stimulation. The levels of phospho-proteins were measured using the Luminex platform. MFI values of each analyte were normalized by GAPDH MFI, and normalized MFI was plotted in the summary boxplot (*: *p*<0.05, **: *p*<0.01). **(B)** Spearman correlation was analyzed between the MFI of CD16 on total NK cells and raw MFI of p-Syk following CD16 stimulation with or without IL-18 and TAPI-1 treatment.
(TIF)
