# Supplementary figures and images for: Multiplex interrogation of the NK cell signalome reveals global downregulation of CD16 signaling during lentivirus infection through an IL-18/ADAM17-dependent mechanism
Source: PLoS Pathog. 2023 Sep 5;19(9):e1011629. doi: 10.1371/journal.ppat.1011629 (PMC10503717; doi:10.1371/journal.ppat.1011629)

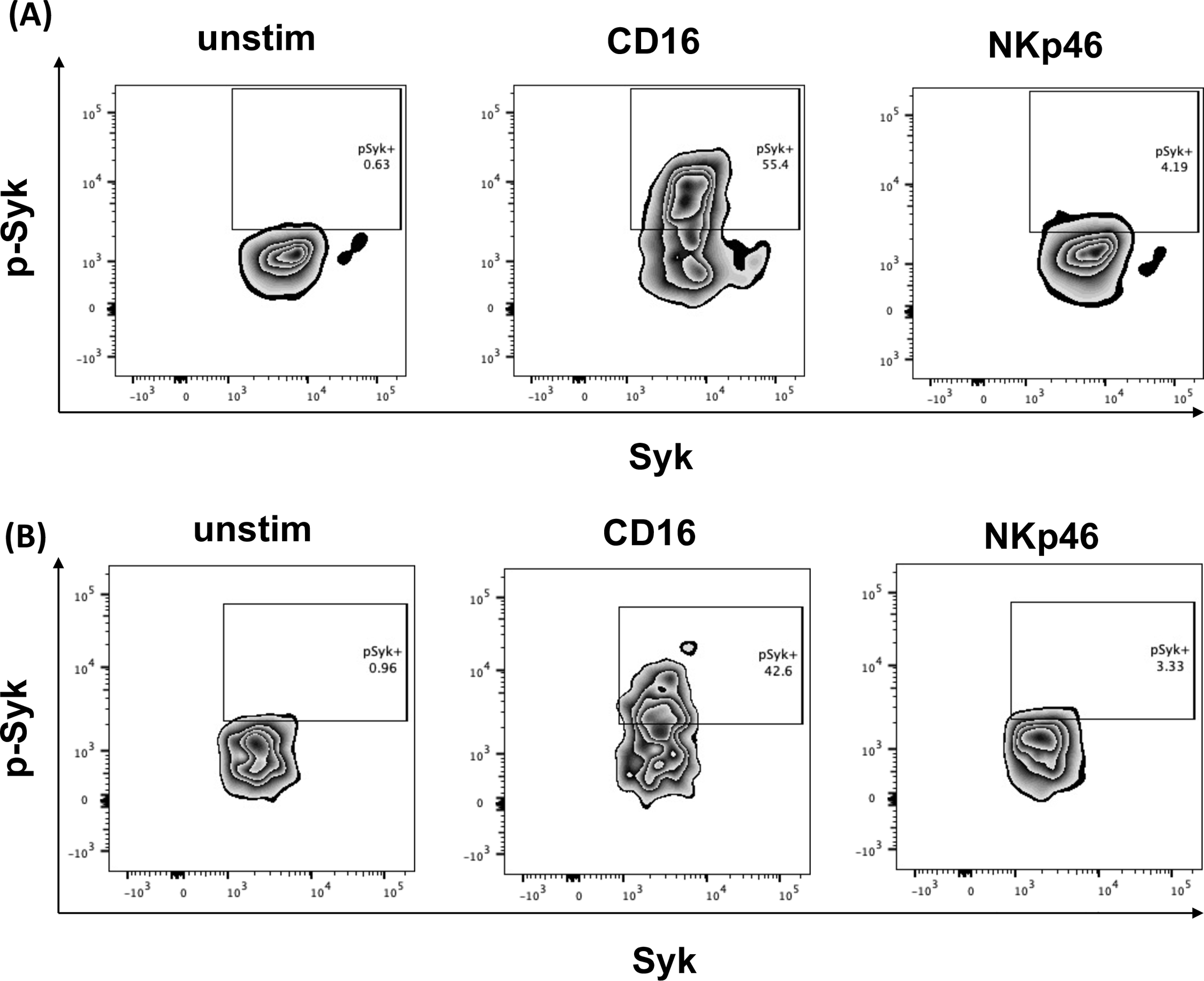

Supplement: S1 Fig — Healthy human PBMC (A) and experimentally naïve RM PBMC (B) were crosslinked with anti-CD16 or NKp46 antibody and the percentages of p-Syk+ cells in live CD3-CD14-CD20-CD56+Syk+ were quantified. Representative flow plots are shown. (TIF) [file ppat.1011629.s002.tif]

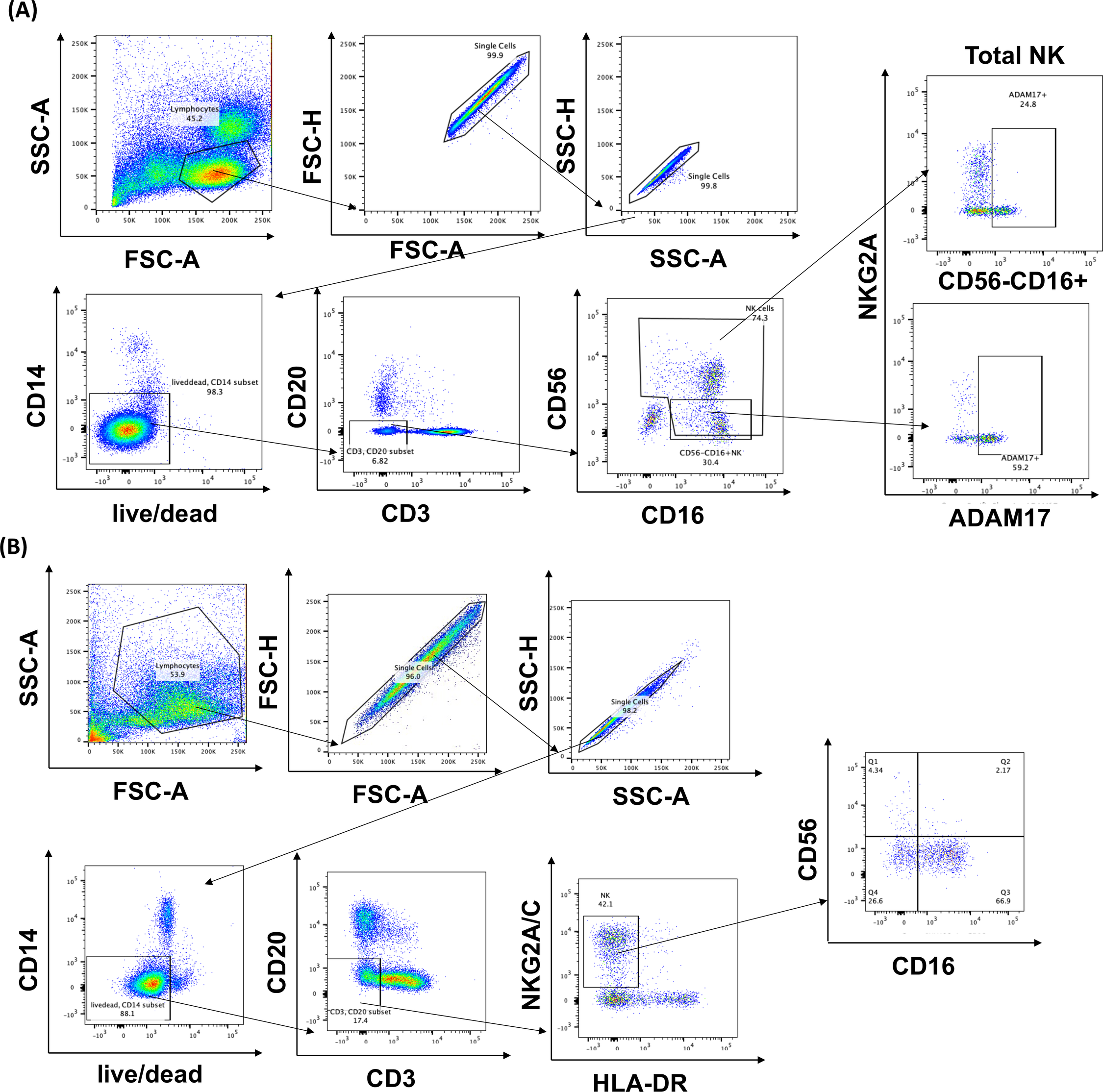

Supplement: S2 Fig — Shown are representative gating strategies to analyze surface and intracellular marker expression on HIV-1-uninfected or PLWH human PBMC (A) and RM PBMC with or without SIV infection (B). (TIF) [file ppat.1011629.s003.tif]

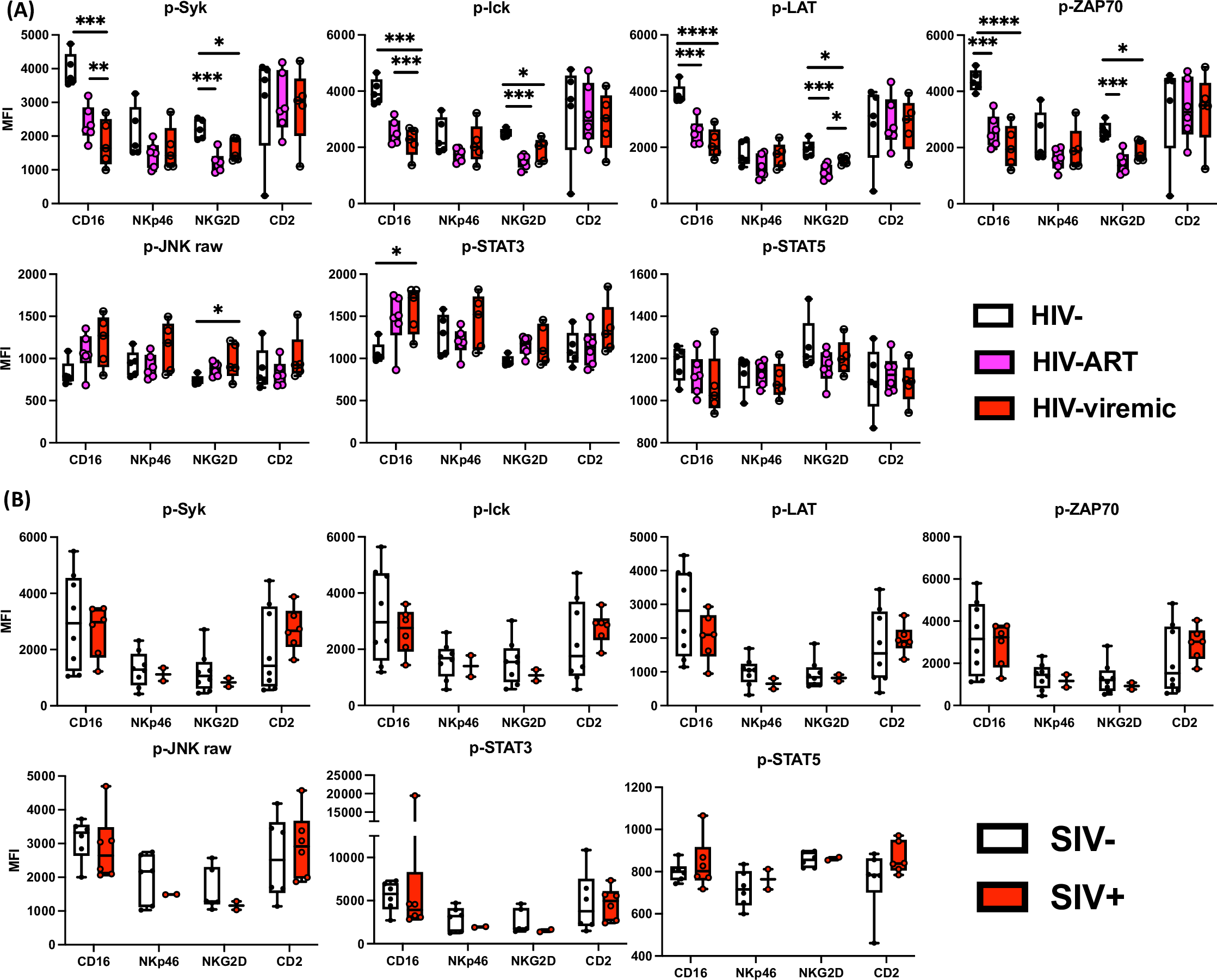

Supplement: S3 Fig — (A) NK cell isolation was performed for PBMC from HIV-1 uninfected individuals (n = 5), PLWH on (n = 6) or off ART (n = 5) and crosslinked with anti-CD16, NKp46, NKG2D, and CD2 antibodies. The levels of phosphorylated proteins were measured by Luminex technology. MFI values of each analyte were normalized by GAPDH MFI, and summary boxplots were shown. (B) NK cells were enriched from naïve (n = 8) and SIV chronically-infected RM (n = 6) and stimulated via CD16, NKp46, NKG2D, and CD2 crosslinking. The amount of phosphorylated analytes was quantified by Luminex platform. normalized MFI by GAPDH levels were plotted in the summary boxplots. (TIF) [file ppat.1011629.s004.tif]

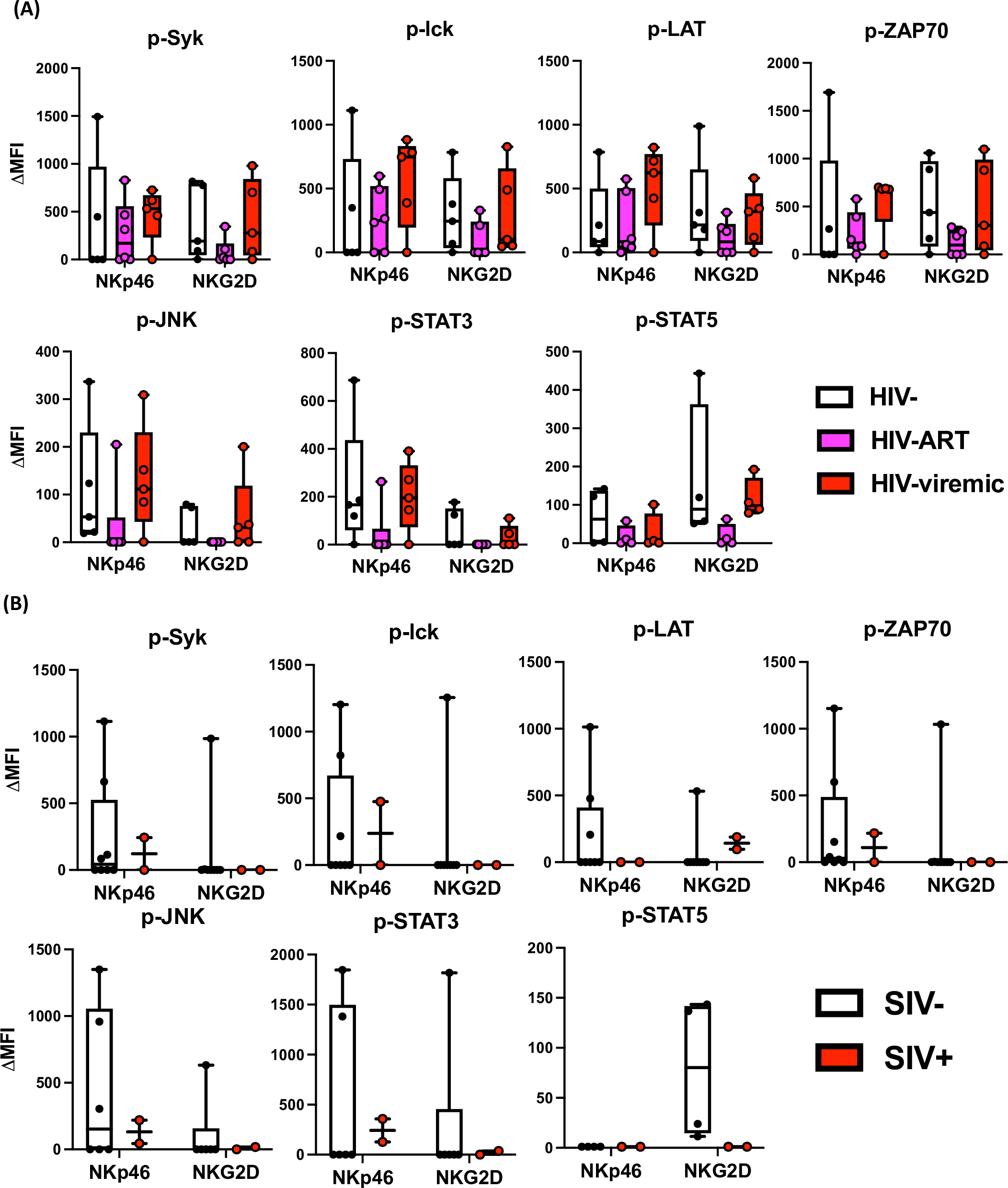

Supplement: S4 Fig — (A) NK cells were purified from PBMC from HIV-1 uninfected subjects (n = 5), PLWH with (n = 6) or without ART (n = 5) and stimulated with NKp46 and NKG2D. the levels of phospho-proteins were measured by Luminex platform. (B) PBMC from SIV-uninfected (n = 8) and chronically infected (n = 2) were enriched for NK cells. Cells were then crosslinked with anti-NKp46 and anti-NKG2D antibodies and the magnitude of phosphorylation of seven signaling molecules was assessed by Luminex assay. ΔMFI was calculated by normalization of raw MFI by GAPDH values followed by background subtraction using β2M- ‘stimulated’ cells. The summary boxplots are depicted, and each dot represents different donors or animals. (TIF) [file ppat.1011629.s005.tif]

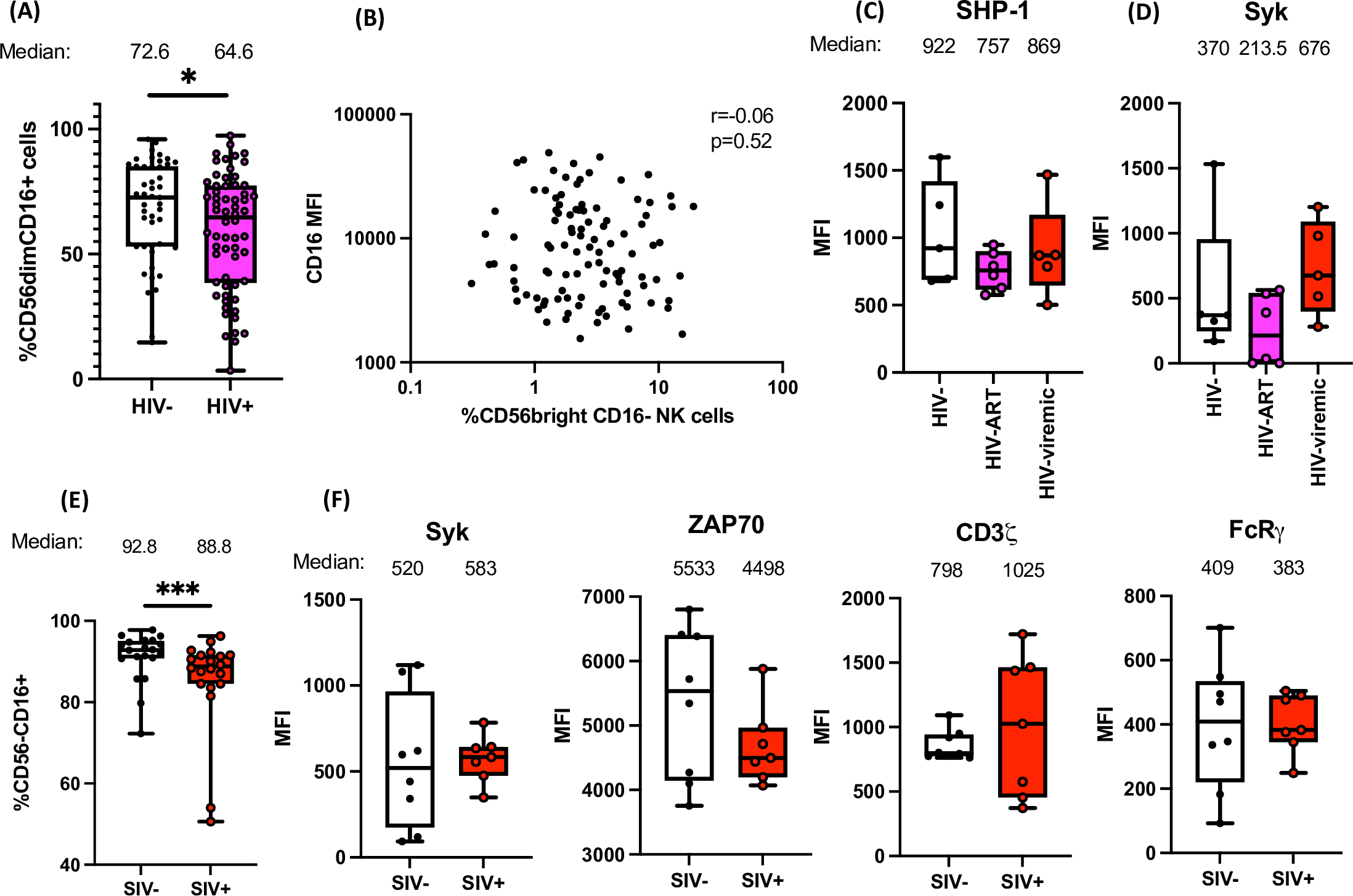

Supplement: S5 Fig — (A) PBMC from HIV-1 uninfected individuals (n = 46), and PLWH receiving ART (n = 58) were stained and %CD56dimCD16+ NK cells of live CD3-CD4-CD14-CD19- were calculated by flow cytometry. (B) The percentages of CD56brightCD16- NK cells were correlated with the expression of CD16 on total NK cells. Spearman correlation coefficient was calculated. (C, D) The levels of SHP-1 (C) and total Syk (D) in total NK cells from HIV-1-uninfected subjects (n = 5), PLWH with (n = 6) or without ART (n = 5) were measured by flow cytometry. (E) PBMC from SIV-uninfected or infected (n = 20) RM were stained and %CD56-CD16+ cells was measured for live CD3-CD14-CD20-HLADR-NKG2A/C+ cells. Each dot represents different animals or subjects. (***: p<0.005). (F) The expression of total Syk, ZAP70, CD3ζ, and FcRγ in total NK cells from SIV-uninfected (n = 7), and chronically infected (n = 6) macaques were quantified by flow cytometry. (TIF) [file ppat.1011629.s006.tif]

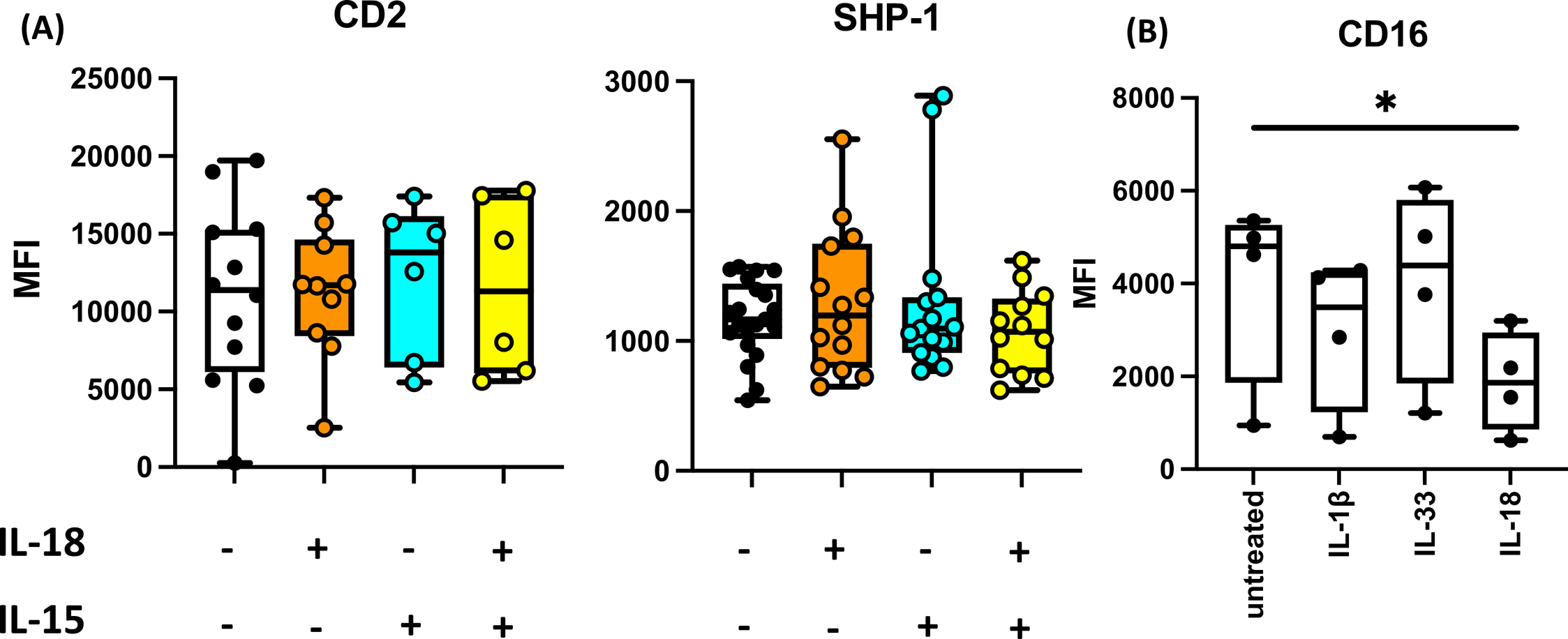

Supplement: S6 Fig — (A) Healthy human PBMC were left untreated (n = 12) or incubated with IL-18 (n = 10), IL-15 (n = 6), or both IL-15 & IL-18 (n = 6). After overnight culture, cells were then stained and analyzed by flow cytometry. The MFI of CD2 and SHP-1 on live total NK cells were quantified. Summary boxplots of CD2 and SHP-1 MFI on live total NK cells were shown, where respective dots represent different subjects. (B) Healthy human PBMC were cultured in the presence or absence of IL-1β, IL-33, and IL-18 overnight and the expression of CD16 on total NK cells were measured by flow cytometry. The statistically significant differences were evaluated using paired t tests. (n = 4, *: p<0.05). (TIF) [file ppat.1011629.s007.tif]

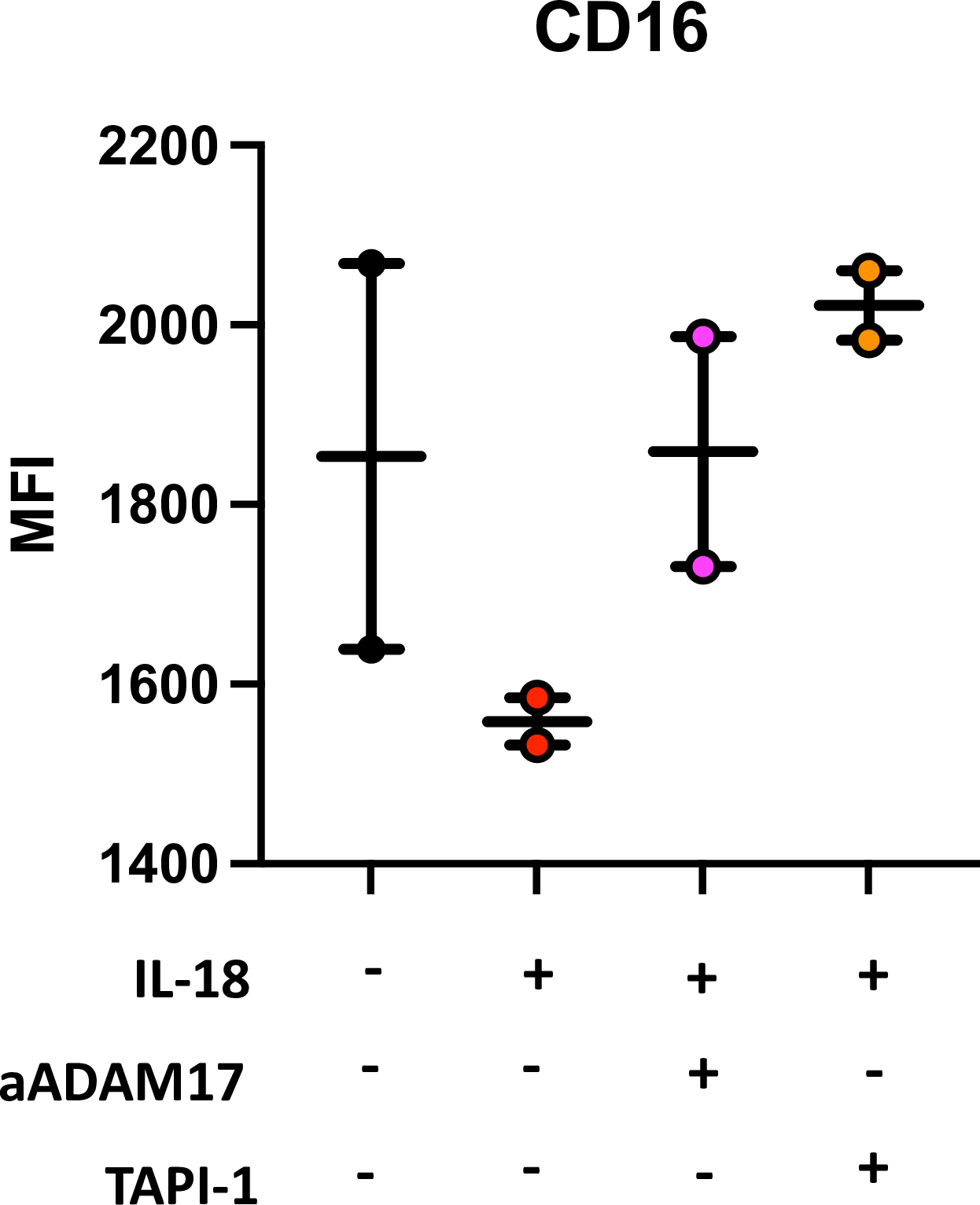

Supplement: S7 Fig — SIV-uninfected RM PBMC were incubated with or without IL-18, ADAM17 blocking antibody (aADAM17), and TAPI-1 (n = 2 for each). Following incubation, cells were stained and flow cytometric analysis was performed to assess the levels of CD16 on NK cells. (TIF) [file ppat.1011629.s008.tif]

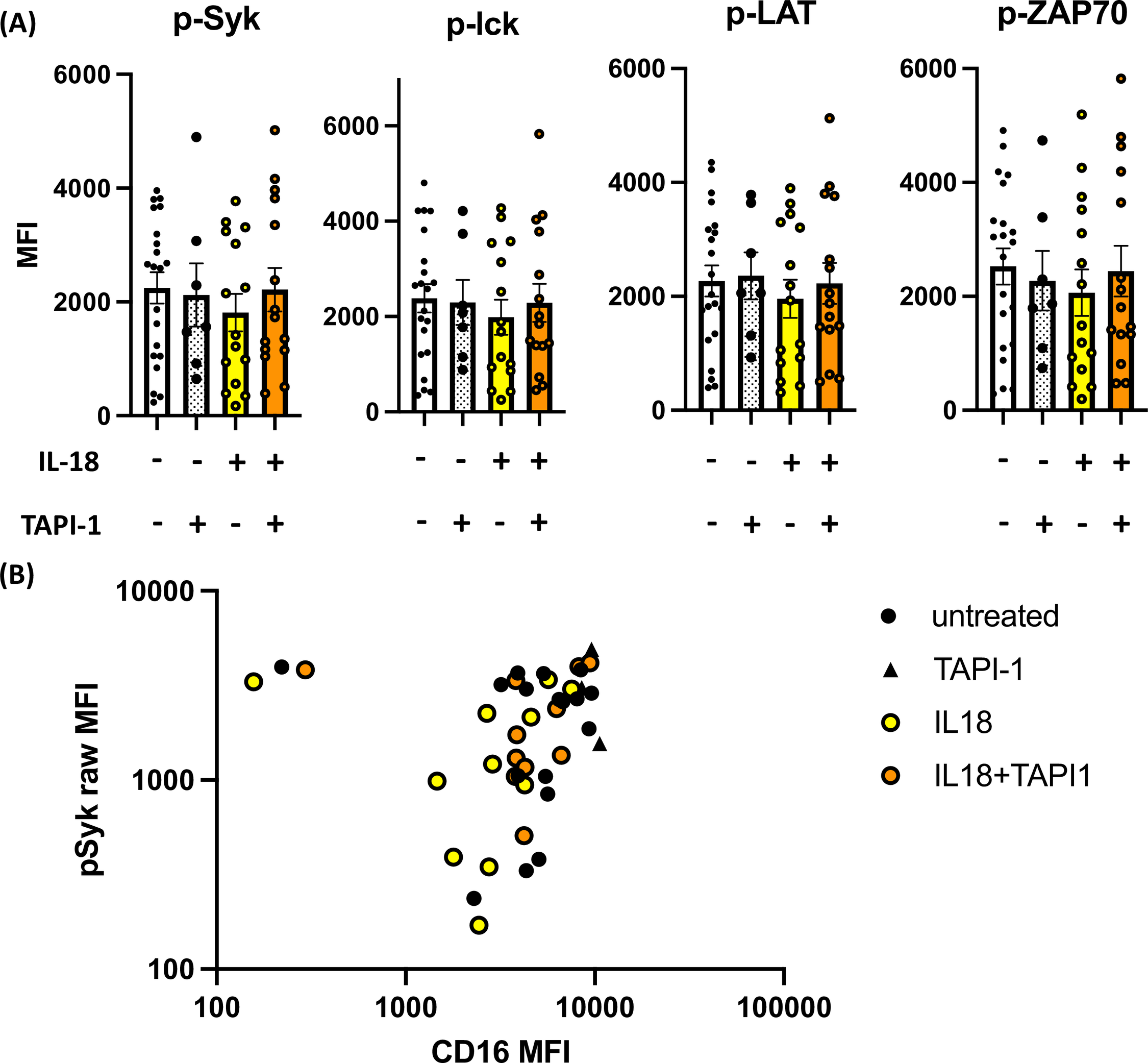

Supplement: S8 Fig — (A) Healthy human PBMC were cultured in the presence or absence of 10ng/mL IL-18 (n = 8) and 25μM TAPI-1 (n = 8) along with IL-15 overnight. NK cells were then isolated and then applied with CD16 stimulation. The levels of phospho-proteins were measured using the Luminex platform. MFI values of each analyte were normalized by GAPDH MFI, and normalized MFI was plotted in the summary boxplot (*: p<0.05, **: p<0.01). (B) Spearman correlation was analyzed between the MFI of CD16 on total NK cells and raw MFI of p-Syk following CD16 stimulation with or without IL-18 and TAPI-1 treatment. (TIF) [file ppat.1011629.s009.tif]
